# A Near‐Infrared Photoactive Morphology Modifier Leads to Significant Current Improvement and Energy Loss Mitigation for Ternary Organic Solar Cells

**DOI:** 10.1002/advs.201800755

**Published:** 2018-06-20

**Authors:** Lingling Zhan, Shuixing Li, Huotian Zhang, Feng Gao, Tsz‐Ki Lau, Xinhui Lu, Danyang Sun, Peng Wang, Minmin Shi, Chang‐Zhi Li, Hongzheng Chen

**Affiliations:** ^1^ State Key Laboratory of Silicon Materials MOE Key Laboratory of Macromolecular Synthesis and Functionalization Department of Polymer Science and Engineering Zhejiang University Hangzhou 310027 P. R. China; ^2^ Biomolecular and Organic Electronics IFM Linköping University Linköping 58183 Sweden; ^3^ Department of Physics Chinese University of Hong Kong New Territories Hong Kong P. R. China; ^4^ Department of Chemistry Zhejiang University Hangzhou 310027 P. R. China

**Keywords:** current enhancement, energy loss, morphology optimization, nonfullerene acceptors, ternary organic solar cells

## Abstract

Herein, efficient organic solar cells (OSCs) are realized with the ternary blend of a medium band gap donor (poly[(2,6‐(4,8‐bis(5‐(2‐ethylhexyl)thiophen‐2‐yl)‐benzo[1,2‐b:4,5‐b′]dithiophene))‐alt‐(5,5‐(1′,3′‐di‐2‐thienyl‐5′,7′‐bis(2‐ethylhexyl)benzo[1′,2′‐c:4′,5′‐c′]dithiophene‐4,8‐dione)] (PBDB‐T)) with a low band gap acceptor (2,2′‐((2Z,2′Z)‐(((2,5‐difluoro‐1,4‐phenylene)bis(4,4‐bis(2‐ethylhexyl)‐4H‐cyclopenta[2,1‐b:3,4‐b′]dithiophene‐6,2‐diyl))bis(methanylylidene))bis(5,6‐difluoro‐3‐oxo‐2,3‐dihydro‐1H‐indene‐2,1‐diylidene))dimalononitrile (HF‐PCIC)) and a near‐infrared acceptor (2,2′‐((2Z,2′Z)‐(((4,4,9,9‐tetrakis(4‐hexylphenyl)‐4,9‐dihydro‐s‐indaceno[1,2‐b:5,6‐b′]dithiophene‐2,7‐diyl)bis(4‐((2‐ethylhexyl)oxy)thiophene‐5,2‐diyl))bis(methanylylidene))bis(5,6‐difluoro‐3‐oxo‐2,3‐dihydro‐1H‐indene‐2,1‐diylidene))dimalononitrile (IEICO‐4F)). It is shown that the introduction of IEICO‐4F third component into PBDB‐T:HF‐PCIC blend increases the short‐circuit current density (*J*_sc_) of the ternary OSC to 23.46 mA cm^−2^, with a 44% increment over those of binary devices. The significant current improvement originates from the broadened absorption range and the active layer morphology optimization through the introduction of IEICO‐4F component. Furthermore, the energy loss of the ternary cells (0.59 eV) is much decreased over that of the binary cells (0.80 eV) due to the reduction of both radiative recombination from the absorption below the band gap and nonradiative recombination upon the addition of IEICO‐4F. Therefore, the power conversion efficiency increases dramatically from 8.82% for the binary cells to 11.20% for the ternary cells. This work provides good examples for simultaneously achieving both significant current enhancement and energy loss mitigation in OSCs, which would lead to the further construction of highly efficient ternary OSCs.

Bulk‐heterojunction organic solar cells (OSCs) have received great progresses in recent years with the development of new photovoltaic materials and device engineering.^1^, ^2^, ^3^, ^4^, ^5^, ^6^, ^7^ In particular with the advantages of strong light‐harvesting capability and ease energy level tunability,^8^, ^9^, ^10^, ^11^, ^12^, ^13^, ^14^ OSCs employing nonfullerene acceptors have made breakthroughs of power conversion efficiencies (PCEs) over 13%.^15^, ^16^, ^17^, ^18^ Though OSCs with the binary blend of a donor and an acceptor as the active layer develop rapidly, there still exist some drawbacks, e.g., limited absorption range for two materials, restricted morphological optimization. To address these issues, ternary OSCs (TOSCs) have been proven to be a simple and effective strategy to further improve the device efficiencies,^19^, ^20^, ^21^, ^22^, ^23^, ^24^, ^25^, ^26^ in which a third component (either as photoactive donor or acceptor or as morphology optimizer) is introduced to the active layers. The third component allows broadening the spectrum coverage or optimizing the morphology, thus promoting the short‐circuit current density (*J*_sc_) or fill factor (FF).^27^, ^28^, ^29^, ^30^, ^31^, ^32^, ^33^, ^34^, ^35^, ^36^, ^37^, ^38^, ^39^, ^40^, ^41^, ^42^, ^43^, ^44^

Although TOSCs are considered as an effective method to promote the performances of OSCs, many challenges remain unresolved for TOSCs. The top one of them is the disrupted morphology of the original binary blend, when the third component is added. As a result, the reduced FF is commonly observed.^45^, ^46^ To make up for the loss in FF, a large enhancement in *J*_sc_ is strongly expected. However, even the photocurrent response is enhanced in the part of the third component due to the broadened absorption, those in the parts of the original donor and acceptor are rarely seen improved, thus limiting the improvement of *J*_sc_ for TOSCs. Consequently, the increases in PCEs of TOSCs are also limited compared to those of binary OSCs. To address this issue, the introduced third component should not only own complementary absorption to those of original donor and acceptor, but also solve the drawbacks in morphology existing in the original binary blend.^21^, ^31^ Therefore, further current and efficiency enhancements may be realized.

Another challenge is how to reduce the energy losses for achieving a high open‐circuit voltage (*V*_oc_) in TOSCs.^8^, ^47^, ^48^, ^49^, ^50^, ^51^, ^52^ For the *V*_oc_, there are three main different situations. The first one is that *V*_oc_ will proportionally vary with the ratios of third component, namely, *V*_oc_ will be lifted by adding a higher LUMO (the lowest unoccupied molecular orbital) acceptor or a deeper HOMO (the highest occupied molecular orbital) donor, and vice versa.^53^, ^54^, ^55^ This situation is the most common one. The second is called the pinning effect, which means the *V*_oc_ is nearly decided by the smallest gap between the HOMO of donor and the LUMO of acceptor regardless of adding a higher LUMO acceptor or a deeper HOMO donor.^56^ This situation is not preferred.^29^ The last one is that *V*_oc_ will almost keep the high value as that of binary OSCs when adding a lower LUMO acceptor.^36^ If constant *V*_oc_ is achieved by adding a third component with near‐infrared absorption and lower LUMO level, energy loss must be reduced to achieve this. Therefore, the last situation is preferred. However, the third situation is still rarely seen in TOSCs. In addition to these three situations, a recent paper reported a TOSC that demonstrated higher *V*_oc_ values than both binary devices.^57^ Although this is an even preferred situation, the mechanism is hardly understood and only one combination has been reported to date.

Here, we report highly efficient fullerene‐free TOSCs that have addressed the above‐mentioned two challenges in current enhancement and energy loss. The TOSCs are based on a polymer donor, poly[(2,6‐(4,8‐bis(5‐(2‐ethylhexyl)thiophen‐2‐yl)‐benzo[1,2‐b:4,5‐b′]dithiophene))‐alt‐(5,5‐(1′,3′‐di‐2‐thienyl‐5′,7′‐bis(2‐ethylhexyl)benzo[1′,2′‐c:4′,5′‐c′]dithiophene‐4,8‐dione)] (PBDB‐T), and two nonfullerene acceptors, 2,2′‐((2Z,2′Z)‐(((2,5‐difluoro‐1,4‐phenylene)bis(4,4‐bis(2‐ethylhexyl)‐4H‐cyclopenta[2,1‐b:3,4‐b′]dithiophene‐6,2‐diyl))bis(methanylylidene))bis(5,6‐difluoro‐3‐oxo‐2,3‐dihydro‐1H‐indene‐2,1‐diylidene))dimalononitrile (HF‐PCIC) and 2,2′‐((2Z,2′Z)‐(((4,4,9,9‐tetrakis(4‐hexylphenyl)‐4,9‐dihydro‐s‐indaceno[1,2‐b:5,6‐b′]dithiophene‐2,7‐diyl)bis(4‐((2‐ethylhexyl)oxy)thiophene‐5,2‐diyl))bis(methanylylidene))bis(5,6‐difluoro‐3‐oxo‐2,3‐dihydro‐1H‐indene‐2,1‐diylidene))dimalononitrile (IEICO‐4F) (**Figure** **1**a). HF‐PCIC is a new nonfullerene acceptor designed in our lab based on our recent published work.^58^ The selection of these three materials to construct TOSCs is based on the following considerations: 1) the absorptions of these three materials are complementary to each other (Figure 1b), especially, due to the well matched absorption ranges of HF‐PCIC and IEICO‐4F, energy transfer may exist between these two nonfullerene acceptors; 2) HF‐PCIC with four 2‐ethylhexyl substituents is a crystalline material easy to aggregate,^58^ while IEICO‐4F with four phenylhexyl substituents is a relatively lower crystalline material harder to aggregate,^9^ the introduction of IEICO‐4F may optimize the domain sizes of PBDB‐T:HF‐PCIC blends; 3) IEICO‐4F is a low energy loss material,^9^ which can reduce the impact of introducing a near‐infrared material with lower LUMO level on *V*_oc_; 4) the *π–π* stacking of acceptor–donor–acceptor (A–D–A) type nonfullerene acceptors is realized through the terminals of molecules, therefore, the same terminal structure of 2‐(5,6‐difluoro‐3‐oxo‐2,3‐dihydro‐1H‐inden‐1‐ylidene)malononitrile (IC‐2F) may allow these two nonfullerene acceptors (HF‐PCIC and IEICO‐4F) to show good compatibility. The ternary PBDB‐T:HF‐PCIC:IEICO‐4F and the control binary PBDB‐T:HF‐PCIC solar cells are fabricated. The optimized TOSCs exhibit the best PCE of 11.20% with an excellent *J*_sc_ of 23.46 mA cm^−2^ and a *V*_oc_ of 0.78 V. Compared with the binary solar cells (PCE: 8.82%, *J*_sc_: 16.26 mA cm^−2^, *V*_oc_: 0.80 V), a dramatic efficiency improvement of 27% and a significant current enhancement of 7.20 mA cm^−2^ are observed in TOSCs. To the best of our knowledge, such a huge current enhancement is the highest value ever reported in TOSCs. Besides, nearly constant *V*_oc_ is achieved regardless of the change of IEICO‐4F ratios. Further characterizations reveal that the energy loss of TOSCs is reduced to 0.59 eV, much smaller than that (0.80 eV) for the binary solar cells. Therefore, our work provides good guidelines for achieving a large current enhancement and a small energy loss in TOSCs, thus leading to the successful construction of highly efficient TOSCs.

**Figure 1:**
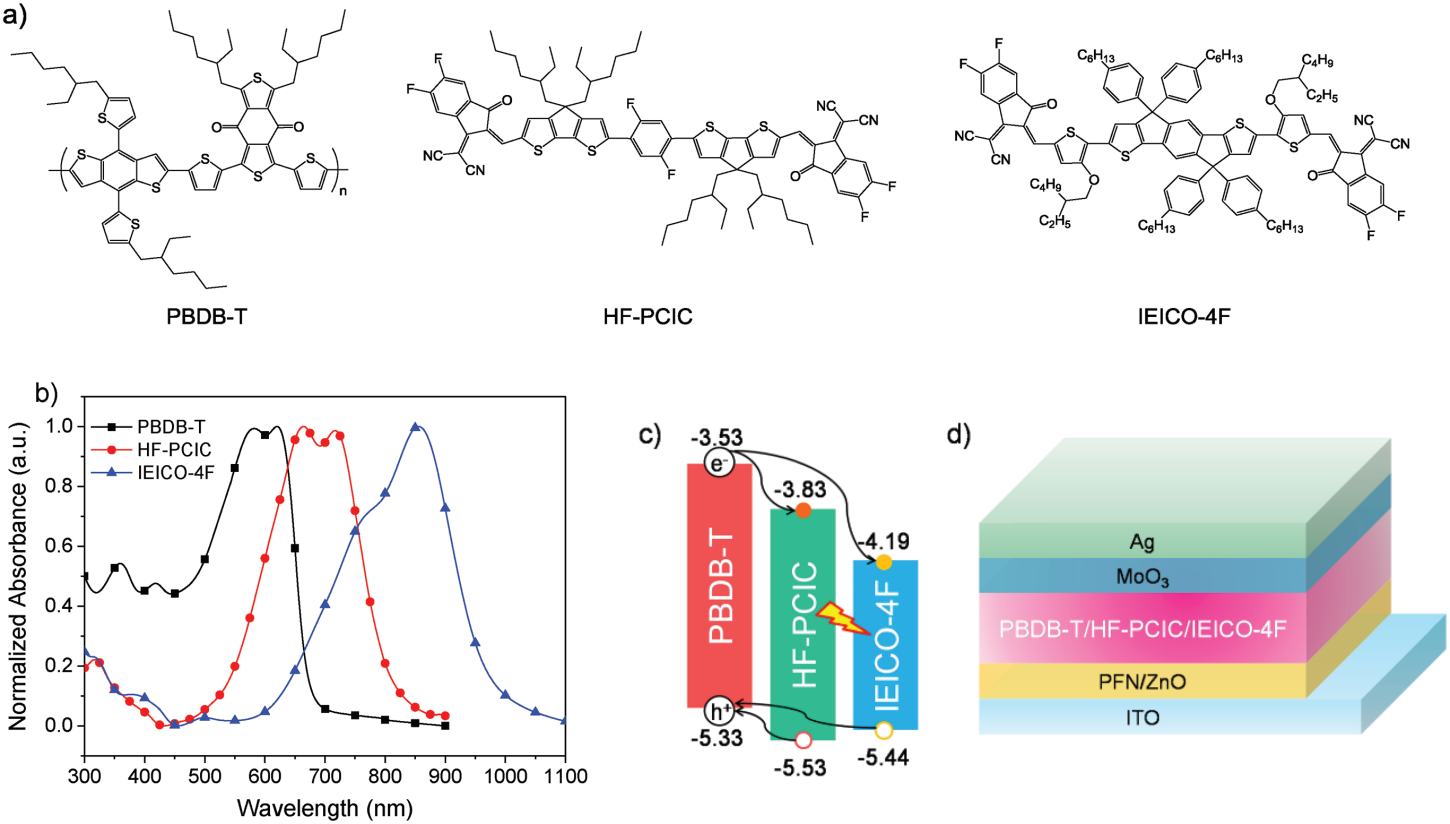
a) Chemical structures of PBDB‐T, HF‐PCIC, and IEICO‐4F. b) UV–vis absorption spectra of PBDB‐T, HF‐PCIC, and IEICO‐4F thin films. c) Energy levels diagram of PBDB‐T, HF‐PCIC, and IEICO‐4F. d) The device structure of ternary organic solar cells.

Figure 1a shows the chemical structures of PBDB‐T, HF‐PCIC, and IEICO‐4F. PBDB‐T is a medium band gap polymer donor widely used in nonfullerene OSCs. Its absorption mainly lies in the range of 300–700 nm. HF‐PCIC acceptor is synthesized in our lab. The detailed information about HF‐PCIC, including the synthetic procedure, thermal properties, and molecular geometry, can be found in the Supporting Information (Figures S1–S4, Supporting Information). From Figure 1b, we can see, the absorption of HF‐PCIC is mainly located in the range of 550–800 nm, which is complementary to that of PBDB‐T. As for IEICO‐4F, it is a near‐infrared acceptor with the absorption extended to near 1000 nm. Therefore, utilizing these three materials to construct TOSCs can realize a broad coverage of absorption from 300 to 1000 nm. The energy levels of these three materials are described in Figure 1c. The LUMO and HOMO of HF‐PCIC are found to be around −3.83 and −5.53 eV. Therefore, a cascade‐type LUMO levels arrangement is formed among these three materials, which is beneficial for exciton dissociation.

An inverted device structure of ITO/ZnO/PFN/Active Layer/MoO_3_/Ag (Figure 1d) is applied to fabricate the OSCs, wherein ITO is indium tin oxide and PFN is poly[9,9‐bis(3′‐(*N,N*‐dimethylamino)propyl)‐2,7‐fluorene)‐*alt*‐2,7‐(9,9‐ioctylfluorene]. We first optimize the binary solar cells based on PBDB‐T:HF‐PCIC blended films. The best performances are obtained with the PBDB‐T:HF‐PCIC weight ratio of 1:1.2, the addition of 0.8% CN (1‐chloronaphthalene) and the thermal annealing at 110 °C for 10 min. More information about the optimization of the binary solar cells can be found in Table S1 (Supporting Information). Keeping the total D/A weight ratio as 1:1.2, different weight ratios of IEICO‐4F are added to the PBDB‐T:HF‐PCIC blends as the third component, and the TOSCs are fabricated with the same post‐treatment methods as those for binary solar cells.

The *J*–*V* curves of binary solar cells and TOSCs are displayed in **Figure** **2**a, and the relevant photovoltaic parameters are summarized in **Table** **1**. The optimized PBDB‐T:HF‐PCIC based binary solar cells can exhibit the best PCE of 8.82% with a *V*_oc_ of 0.80 V, a *J*_sc_ of 16.26 mA cm^−2^, and an FF of 68.44%. When 25% IEICO‐4F is introduced as the third component, the PCE of TOSCs is improved to 10.41% with a *V*_oc_ of 0.79 V, a *J*_sc_ of 20.34 mA cm^−2^, and an FF of 63.01%. It is obvious that the addition of IEICO‐4F has little impact on the *V*_oc_ and a negative effect on the FF. However, the *J*_sc_ has a huge enhancement of 4.08 mA cm^−2^, thus promoting the improvement of PCE. Upon the addition of 35% IEICO‐4F to the ternary blends, a champion PCE of 11.20% is achieved. For the champion device, though the FF is further reduced to 60.99%, the *J*_sc_ shows a recorded value of 23.46 mA cm^−2^, which is responsible for the substantial improvement of PCE. Notably, compared with the PBDB‐T:HF‐PCIC based binary solar cells, a significant current enhancement of 7.2 mA cm^−2^ is observed. If further increasing the weight ratio of IEICO‐4F to 45%, the PCE is reduced to 9.93% with the decrease in *J*_sc_ and FF. We also fabricate the binary solar cells based on PBDB‐T:IEICO‐4F blends under the same conditions as those for PBDB‐T:HF‐PCIC based devices. The PCE of PBDB‐T:IEICO‐4F based devices is relatively low with the value of 6.64% due to the disappointing FF of 44.60%. The above results reveal that IEICO‐4F is a satisfied material as the third component to enhance the *J*_sc_ for TOSCs. More results of ternary solar cells with different weight ratios of IEICO‐4F can be found in Table S2 (Supporting Information), and stable voltages as high as 0.76 V are still remained even if the weight ratios of IEICO‐4F vary from 55% to 85%.

**Figure 2:**
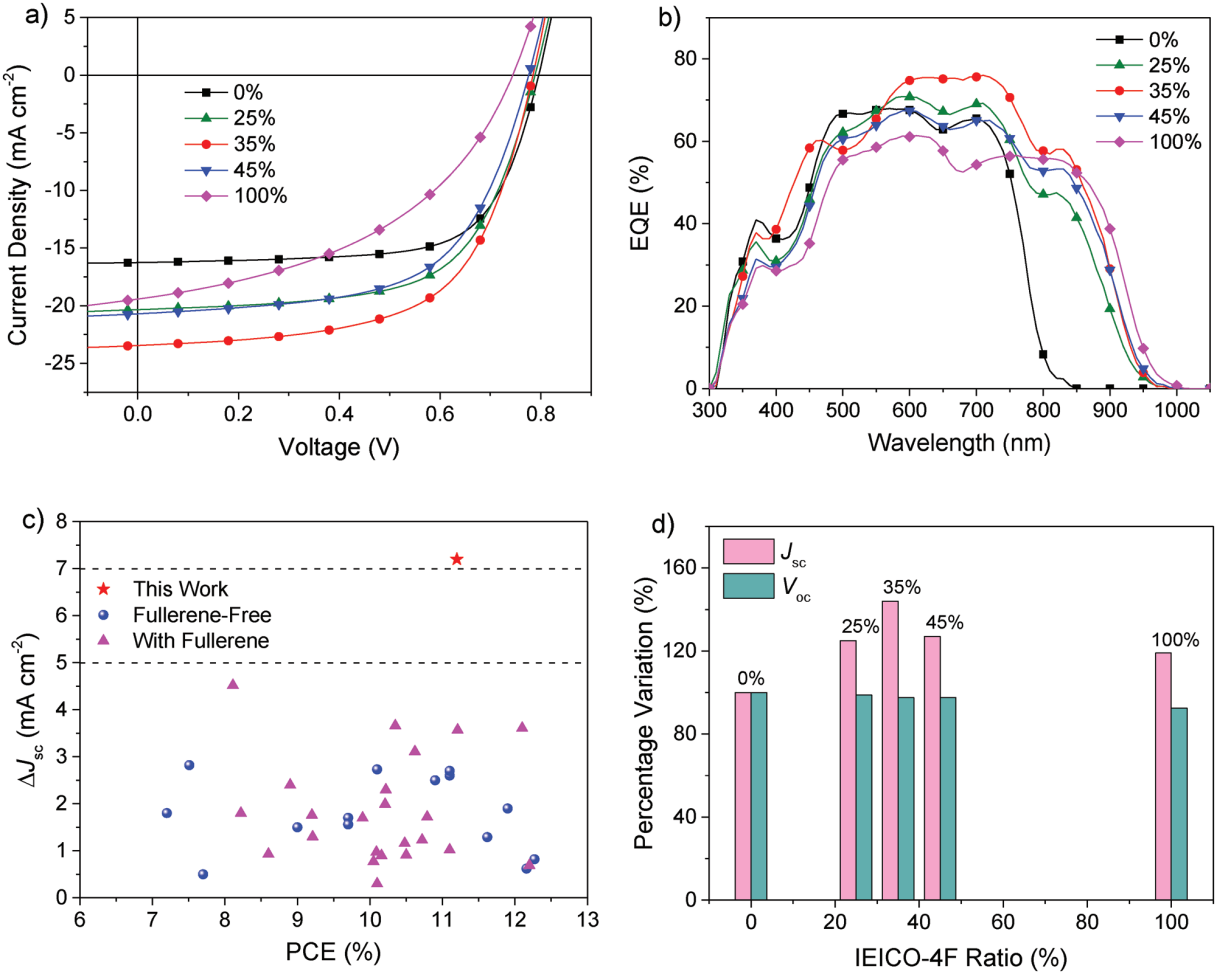
a) *J*–*V* curves of the OSCs with different weight ratios of IEICO‐4F. b) EQE curves of the corresponding OSCs. c) Comparison of current enhancements and PCEs of our work and reported ternary solar cells (see Table S5, Supporting Information). d) The percentage variation of *J*_sc_ and *V*_oc_ with the change of IEICO‐4F ratio (0%, 25%, 35%, 45%, and 100%).

**Table 1:** Photovoltaic parameters of OSCs with different weight ratios of IEICO‐4F

IEICO‐4F ratiosa)	*V*_oc_ [V]	*J*_sc_ [mA cm^−2^]	FF [%]	PCE_max_ [%]	PCE_avg._ b) [%]	*J*_calc_ c)
0%	0.80	16.26	68.44	8.82	8.73	15.52
25%	0.79	20.34	63.01	10.41	10.38	19.12
35%	0.78	23.46	60.99	11.20	11.13	21.39
45%	0.78	20.71	60.02	9.93	9.89	19.19
100%	0.74	19.43	44.60	6.64	6.57	18.22

^a)^ The weight ratios of IEICO‐4F in the acceptor mixture, and the total D/A weight ratio is fixed as 1:1.2

^b)^ Average PCEs for ten devices

^c)^ Calculated *J*_sc_ from the corresponding EQE curves.

External quantum efficiency (EQE) spectra are presented in Figure 2b. Compared with PBDB‐T:HF‐PCIC based binary solar cells, all the TOSCs based on PBDB‐T:HF‐PCIC:IEICO‐4F blends show a broader photocurrent response in the range of 300–1000 nm. Without doubts, the photocurrent response of TOSCs beyond 800 nm is mainly from the contribution of IEICO‐4F, which leads to the major *J*_sc_ enhancement in TOSCs. More importantly, it is observed that the photocurrent responses are also enhanced in the ranges of 400–500 and 550–800 nm. Since PBDB‐T and HF‐PCIC are responsible for the photocurrent responses in the ranges of 400–700 nm and 550–800 nm, respectively, the addition of IEICO‐4F in fact helps improve the photocurrent response of both PBDB‐T and HF‐PCIC. Besides, the device with 35% IEICO‐4F shows the highest EQEs in all of main absorption bands from the three components, implying that synergistic effect of three components induces the optimized morphology, which is in accordance with the results in *J*–*V* curves.

As shown in Figure 2c, we summarize and compare the current enhancement between our work and some reported TOSCs. It can be seen from Figure 2c that all the reported TOSCs can only achieve a current enhancement below 5 mA cm^−2^ and mostly in the range of 1–3 mA cm^−2^. The current enhancement of 7.2 mA cm^−2^ observed in our work is the highest value ever seen so far in TOSCs. Such a large current enhancement corresponds to a 44% improvement (Figure 2d). Besides, nearly constant *V*_oc_ is observed regardless of the change of IEICO‐4F ratios. Since IEICO‐4F is a near‐infrared material with lower LUMO, the negligible loss in voltage is rare and preferred for achieving highly efficient TOSCs.

To explore the causes of the huge current enhancement, we measure the steady photoluminescence (PL) spectra of acceptor mixtures with different weight ratios of IEICO‐4F (**Figure** **3**a). For the neat HF‐PCIC film, its PL spectrum starts from 800 nm and ends at around 1200 nm with a peak at 955 nm. Such PL spectrum of HF‐PCIC is overlapped with the absorption of IEICO‐4F in the range of 800–1000 nm, indicating that energy transfer from HF‐PCIC to IEICO‐4F may exist. Besides, the intensity of PL emission from HF‐PCIC is extremely strong with the highest value of around 8 × 10^6^. As for the neat IEICO‐4F film, its PL spectrum locates in the range of 900–1200 nm with a weak peak (intensity: around 1 × 10^6^) at 975 nm. When IEICO‐4F is added to HF‐PCIC, the PL emission of HF‐PCIC is instantly diminished while the PL emission of IEICO‐4F is significantly enhanced with the intensity two times over that of the neat IEICO‐4F, suggesting that efficient energy transfer happens between HF‐PCIC and IEICO‐4F (Figure 1c). PL spectra of two binary blends and one ternary blend are also measured (Figure S5, Supporting Information). It is shown that the quenching of ternary blend is more sufficient than two binary blends, illustrating that exciton dissociation is more efficient for the ternary blend.

**Figure 3:**
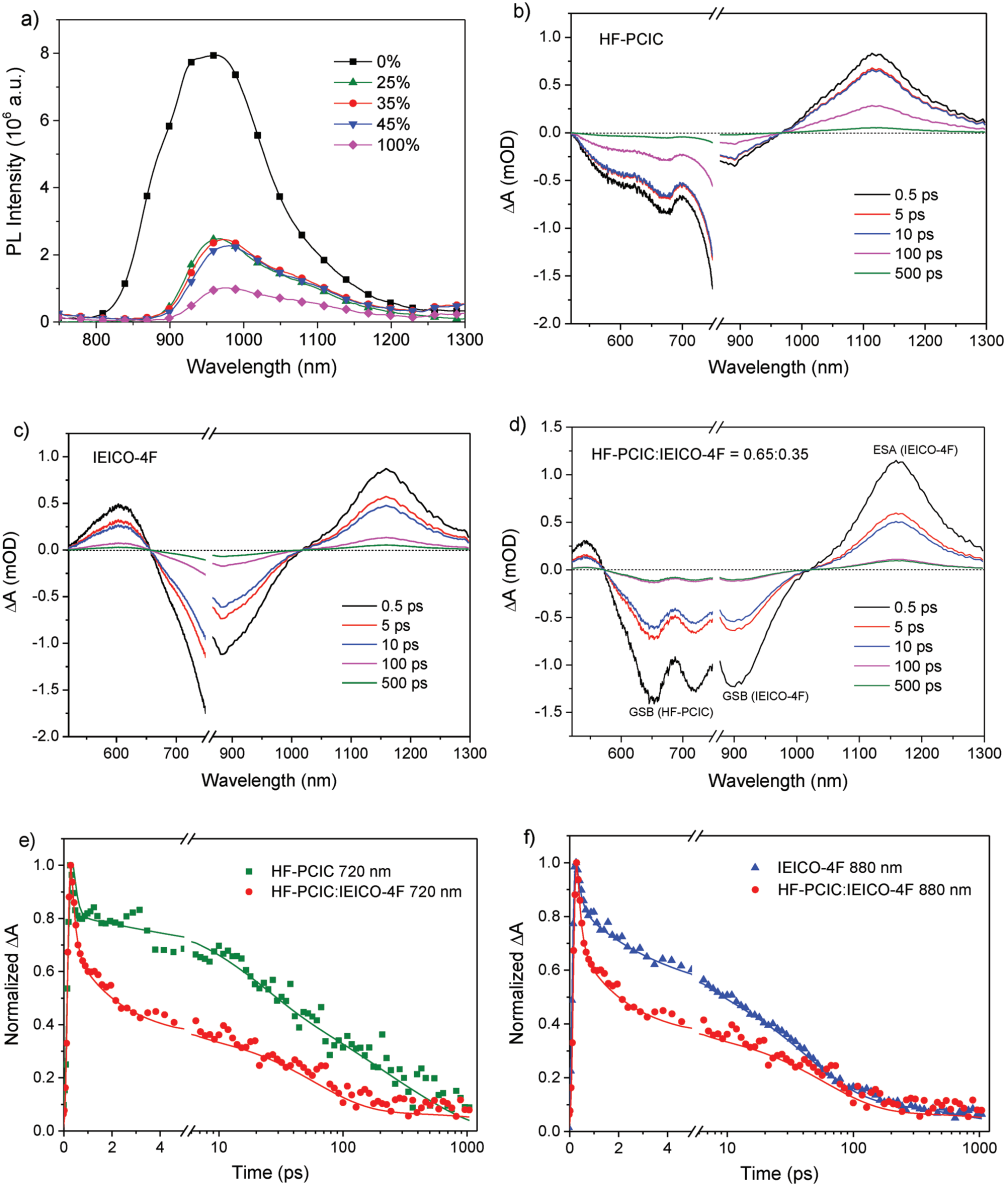
a) Photoluminescence spectra of HF‐PCIC:IEICO‐4F blended films of various ratios excited at 680 nm. b) Transient absorption (TA) spectra of neat HF‐PCIC film. c) TA spectra of neat IEICO‐4F film. d) TA spectra of blended HF‐PCIC:IEICO‐4F film with 35% IEICO‐4F. e) The kinetic curves of the neat HF‐PCIC and HF‐PCIC:IEICO‐4F blended films probed at 720 nm. f) The kinetic curves of the neat IEICO‐4F and HF‐PCIC:IEICO‐4F blended films probed at 880 nm.

We further confirm the energy transfer via the transient absorption (TA) spectroscopy measurements, and the results are shown in Figure 3b–d. Two main features of ground‐state bleaching (GSB) and excited‐state absorption (ESA) are observed in TA spectra. The GSB region mainly reflects the absorption of electron transition from ground‐state to excited state, while ESA stands for the absorption of excitons at excited state. For the neat HF‐PCIC film, GSB signals show a weak peak at 673 nm and a strong peak near 750 nm, which are close to the major absorption band of HF‐PCIC, and ESA signals locate in the near‐infrared range of 965–1300 nm with a peak centered at 1120 nm. For the neat IEICO‐4F, GSB signals show a main peak at 885 nm, which is also corresponded with IEICO‐4F's main absorption band, and ESA signals lie in the near‐infrared range of 1020–1300 nm with a peak centered at 1160 nm. When HF‐PCIC is blended with IEICO‐4F, three peaks appear in GSB signals. The peaks centered at 654 and 720 nm belong to HF‐PCIC, while another peak centered at 900 nm belongs to IEICO‐4F. It is obvious that the intensity of the peak at 720 nm is reduced strongly when compared with that in neat HF‐PCIC film. In return, ESA signals are dominated by those of IEICO‐4F and the intensities of ESA (IEICO‐4F) signals are enhanced, indicating that the energy of HF‐PCIC is efficiently transferred into IEICO‐4F. To ascertain whether the addition of polymer donor PBDB‐T will hinder the energy transfer from HF‐PCIC to IEICO‐4F, we also perform the TA spectroscopy measurements of binary and ternary blends and find similar GSB, ESA changes, and energy transfer phenomenon as those without PBDB‐T (Figure S6, Supporting Information).

Figure 3e,f shows the kinetic curves of neat HF‐PCIC, neat IEICO‐4F, and HF‐PCIC:IEICO‐4F blended films probed at 720 or 880 nm. The kinetic curves probed at 720 nm can more pertinently compare the signals from HF‐PCIC while the kinetic curves probed at 880 nm can compare the signals from IEICO‐4F. Every kinetic curve can be fitted with four lifetime constants, therefore four lifetime constants are obtained for neat HF‐PCIC from green line in Figure 3e, another four lifetime constants are obtained for neat IEICO‐4F from blue line in Figure 3f. As for HF‐PCIC:IEICO‐4F blended film, the four lifetime constants are fitted from kinetic curves probed at 720 or 880 nm. The relevant results are listed in Table S3 (Supporting Information). At the early stage, τ_1_ represents the excitation process with the time scale of less than 1 ps. Here, in our cases, the values of τ_1_ are 0.14 and 0.18 ps for the neat HF‐PCIC and IEICO‐4F films, respectively. Then, τ_2_ reflects the first decay stage with the time scale of 10 ps, more or less. In our cases, the values of τ_2_ are 18.76 and 3.18 ps for the neat HF‐PCIC and IEICO‐4F films, respectively. When HF‐PCIC is mixed with IEICO‐4F, it can be seen from the kinetic curves that the excitation process is not influenced (τ_1_ = 0.15 ps), but the first decay stage becomes faster (τ_2_ = 1.95 ps). We suppose, the shortened lifetime constant of the first decay stage indicates the possible existence of extra process inducing the rapid decay besides spontaneous quenching. This extra process could be the energy transfer between HF‐PCIC and IEICO‐4F, and this process of energy transfer should also be fast. Apparently, energy transfer between HF‐PCIC and IEICO‐4F confirmed by PL and TA spectroscopy measurements plays an important role in the achievement of significant current enhancement, especially in the photocurrent response of IEICO‐4F.

The space charge limited current (SCLC) method is applied to check the charge transport properties of PBDB‐T:HF‐PCIC:IEICO‐4F films with various compositions (Figure S8, Supporting Information), and relevant data are summarized in Table S4 (Supporting Information). The structures of hole‐only devices are ITO/PEDOT:PSS/Active Layer/MoO_3_/Ag, in which PEDOT:PSS is poly(3,4‐ethylenedioxythiophene):poly(styrenesulfonate), while those of electron‐only devices are ITO/ZnO/PFN/Active Layer/PFN/Al. For the binary devices, the hole mobilities and electron mobilities are measured to be 1.63 × 10^−4^ cm^2^ V^−1^ s^−1^ and 0.47 × 10^−4^ cm^2^ V^−1^ s^−1^ for PBDB‐T:HF‐PCIC films, and 3.88 × 10^−4^ cm^2^ V^−1^ s^−1^ and 0.19 × 10^−4^ cm^2^ V^−1^ s^−1^ for PBDB‐T:IEICO‐4F films, respectively. After IEICO‐4F is introduced to PBDB‐T:HF‐PCIC films, all the ternary devices show improved hole mobilities but reduced electron mobilities when compared with those of PBDB‐T:HF‐PCIC films. The improved hole mobilities can help enhance the photocurrent response of PBDB‐T. Anyway, among the ternary devices, the film with 35% IEICO‐4F possesses the highest values and the most balanced ratio of hole and electron mobilities, thus allowing TOSCs with 35% IEICO‐4F show the best performances.

Grazing‐incidence wide‐angle X‐ray scattering^59^, ^60^, ^61^ (GIWAXS) characterization is applied to explore the variations of crystallinity and molecular orientation in binary and ternary films (**Figure** **4**). Neat PBDB‐T film shows a (100) lamellar diffraction peak at 0.300 Å^−1^ both in the out‐of‐plane and in‐plane directions, but the intensity of the peak in the out‐of‐plane direction is much stronger than that in the in‐plane direction. Besides, a weak broad (010) *π–π* stacking diffraction peak appears at 1.67 Å^−1^ in the out‐of‐plane direction, giving a stacking distance of 3.76 Å. For the neat HF‐PCIC film, a lamellar peak appears in the in‐plane direction at 0.490 Å^−1^ and a strong *π–π* stacking peak appears in the out‐of‐direction at 1.73 Å^−1^ (*d* = 3.63 Å), indicating that HF‐PCIC crystalline domains are dominantly face‐on oriented. Along the *q*_z_ axis, there is another peak at 0.680 Å^−1^, indicating the existence of an additional structural ordering (*d* = 9.38 Å) normal to the substrate for the HF‐PCIC crystalline packing, though its origin is not clear. For the neat IEICO‐4F film, a *π–π* stacking peak at 1.81 Å^−1^ in the out‐of‐plane direction, giving a stacking distance of 3.47 Å, and a lamellar peak at 0.300 Å^−1^ in the in‐plane direction are observed, illustrating that IEICO‐4F also prefers the face‐on orientation. The binary PBDB‐T:HF‐PCIC blended film shows the in‐plane lamellar peak at 0.300 Å^−1^ and the out‐of‐plane lamellar peak at 0.330 Å^−1^, originated from the PBDB‐T crystalline domains. Compared with the corresponding peak intensity observed in the neat PBDB‐T film, the face‐on ordering is enhanced while the edge‐on ordering is weakened, which is a good trend for hole transport in photovoltaic devices. The scattering peaks of HF‐PCIC crystalline domains (in‐plane: 0.490 Å^−1^; out‐of‐plane: 0.680 and 1.73 Å^−1^) can be clearly observed, indicating the maintaining of the HF‐PCIC crystalline packing in the binary film. When 25% IEICO‐4F is introduced to PBDB‐T:HF‐PCIC blended film, all the peaks remain at the same position, except for the *π–π* stacking peak, which appears at a relatively larger *q* (≈1.76 Å^−1^) in the out‐of‐plane. When the content of IEICO‐4F is increased to 35%, a similar GIWAXS pattern is observed. If further increasing the content of IEICO‐4F to 45%, the scattering intensity is significantly weakened, suggesting a decrease of the overall crystallinity of the film, which is not preferred. In a word, the enhancement of face‐on ordering and the tighter *π–π* stacking when not too much IEICO‐4F was added should be responsible for the photocurrent response enhancement of PBDB‐T and HF‐PCIC, compared with the PBDB‐T:HF‐PCIC based binary solar cells.

**Figure 4:**
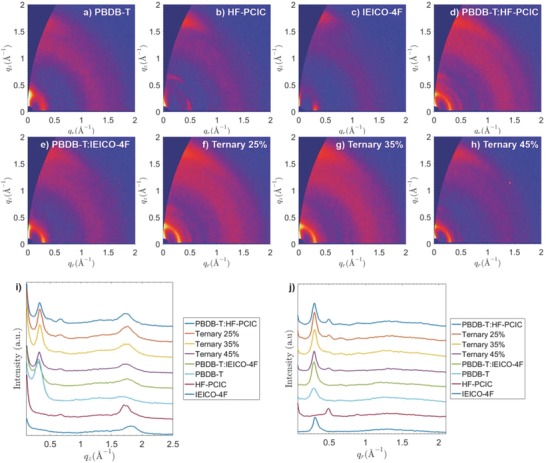
The 2D GIWAXS images of a) PBDB‐T, b) HF‐PCIC, c) IEICO‐4F, d) PBDB‐T:HF‐PCIC, e) PBDB‐T:IEICO‐4F, f) Ternary 25%, g) Ternary 35%, and h) Ternary 45%. The 1D X‐ray profiles along i) *q*_z_ axis and j) *q*_r_ axis of the corresponding thin films.

To investigate the impact of introducing IEICO‐4F on the thin film morphology, atomic force microscopy (AFM) and transmission electron microscopy (TEM) measurements are performed (Figure S9, Supporting Information). Totally different morphological features are observed for two binary thin films in AFM images. A rough surface with obvious domains is present for the PBDB‐T:HF‐PCIC film, while the PBDB‐T:IEICO‐4F film shows a smooth and homogeneous surface. These should be related with the high crystalline property of HF‐PCIC and the relative low crystalline nature of IEICO‐4F. When IEICO‐4F is added to the PBDB‐T:HF‐PCIC films, the obtained ternary blends exhibit decreased surface roughness with reduced domain sizes. This change is reasonable since the low crystalline IEICO‐4F will influence the crystallization behavior of HF‐PCIC. But if too much IEICO‐4F is added, charge transport of the blends will be hindered, just as demonstrated in the SCLC measurement. Therefore, ternary blend with 35% IEICO‐4F shows the balanced domain sizes with superior charge transport properties remained. TEM images show the similar trend as revealed in AFM images. The optimized domain sizes, thereby a better morphology should be responsible for the photocurrent enhancement of HF‐PCIC.

In addition to the advantage of obtaining high *J*_sc_, employing nonfullerene acceptors in OSCs is an effective way to lower the energy loss, thus achieving a high *V*_oc_. Normally, energy loss can be defined as the following equation (see Note 1 in the Supporting Information)^62^, ^63^

(1) $$E_{Loss}\left(\Delta E\right)=E_{gap}-qV_{oc}=\left(E_{\text{gap}}-qV_{\text{oc}}^{\text{SQ}}\right)+\left(qV_{\text{oc}}^{\text{SQ}}-qV_{\text{oc}}^{\text{rad}}\right)+\left(qV_{\text{oc}}^{\text{rad}}-qV_{\text{oc}}\right)=\left(E_{\text{gap}}-qV_{\text{oc}}^{\text{SQ}}\right)+q\Delta V_{\text{oc}}^{\text{red},\text{below}\;\text{gap}}+q\Delta V_{\text{oc}}^{\text{non‐rad}}=\Delta E_{1}+\Delta E_{2}+\Delta E_{3}$$

where *E*_loss_ or Δ*Ε* is the energy loss, *E*_gap_ is the band gap, *V*_oc_^SQ^ is the maximum voltage calculated by the Shockley–Queisser limit, *V*_oc_^rad^ is the open‐circuit voltage when there is only radiative recombination, Δ*V*_oc_^rad,below gap^ is the voltage loss of radiative recombination from the absorption below the band gap, and Δ*V*_oc_^non‐rad^ is the voltage loss of nonradiative recombination. Therefore, energy loss (Δ*Ε*) consists of three parts (Δ*Ε*_1_, Δ*Ε*_2_, and Δ*Ε*_3_). In general, Δ*Ε*_1_ (*E*_gap_ – *qV*_oc_^SQ^) is unavoidable and the value could be 0.25–0.30 eV. As for Δ*Ε*_2_ (*q*Δ*V*_oc_^rad,below gap^), it varies greatly among different types of solar cells. It is negligible for inorganic or perovskite solar cells, but usually high for OSCs. In OSCs, a large contribution to Δ*Ε*_2_ is the existence of charge‐transfer (CT) states. The third part of Δ*Ε*_3_ (*q*Δ*V*_oc_^non‐rad^) can be calculated through the following equation

(2) $$qV_{\mathrm{oc}}^{\mathrm{non‐rad}}\;=\;-kT\ln\left({\mathrm{EQE}}_{\mathrm{EL}}\right)$$

where *k* is the Boltzmann constant (≈1.38 × 10^−23^ *J/K*), *T* is the absolute temperature, and EQE_EL_ is the radiative quantum efficiency of the solar cell when charge carriers are injected into the device in the dark.

To confirm the origin of nearly constant *V*_oc_ and find out the exact values of three parts of energy loss, we measure the Fourier‐transform photocurrent spectroscopy external quantum efficiency (FTPS‐EQE) (**Figure** **5**a) and electroluminescence (EL) spectra (Figure 5b) of two binary devices (0% and 100% blends) and one ternary device (35% blend). Here, to obtain the optical gaps of these devices, we use the method proposed by Uwe Rau (see Note 2 in the Supporting Information).^64^ In this method, the distributions of *E*_gap_ are obtained by taking the derivative of EQE edges (Figure S10, Supporting Information), and then the products of *E*_gap_'s distribution and *E*_gap_ itself are integrated to calculate the specific optical *E*_gap_ for 0%, 35%, and 100% blend‐based devices, and the results are listed in **Table** **2**. From FTPS‐EQE spectra, we can see that the curve of 35% blend overlaps with that of 100% blend well, but differs from that of 0% blend. From EL curves, we can find that a broad EL curve is presented for 0% blend‐based device and the highest peak also locates at an obvious red‐shifting position relative to the band gap, indicating obvious CT state exists in 0% blend‐based device. As for the CT wavelength, we can find it in the curves of TA spectra for PBDB‐T:HF‐PCIC blend (Figure S6b, Supporting Information) and it is shown that the CT state centers at around 900 nm, corresponding to 1.38 eV. Since the EQE_EL_ values (see Note 3 in the Supporting Information) are measured to be 4.3 × 10^−7^, 2.7 × 10^−5^, and 1.6 × 10^−5^ for 0%, 35%, and 100% blends, respectively, Δ*Ε*_3_ values are calculated as 0.38, 0.28, and 0.28 eV for 0%, 35%, and 100% blends. Obviously, the nonradiative recombination is greatly reduced for TOSCs, relative to that of PBDB‐T:HF‐PCIC‐based binary solar cells. Besides, Δ*Ε*_2_ values are found to be 0.13, 0.04, and 0.04 eV for 0%, 35%, and 100% blends, respectively, which means the radiative recombination from the absorption below the band gap is also largely reduced for TOSCs, relative to that of PBDB‐T:HF‐PCIC‐based binary solar cells. Such a low Δ*Ε*_2_ value of 0.04 eV for TOSCs is comparable to that of inorganic solar cells. Without doubts, the decreases of Δ*Ε*_2_ and Δ*Ε*_3_ lead to the reduced energy loss of 0.59 eV for TOSCs. Interestingly, by adding IEICO‐4F to the binary blend, the energy loss of ternary blend is directly determined by the material with the lowest energy loss, which is preferred. So, the achievement of a small energy loss of 0.59 eV should be the reason for the nearly constant *V*_oc_ in TOSCs.

**Figure 5:**
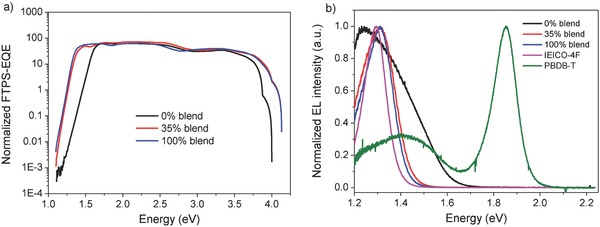
a) Normalized FTPS‐EQE spectra of 0%, 35%, and 100% blend‐based devices. b) Normalized EL curves of 0%, 35%, and 100% blends, IEICO‐4F and PBDB‐T‐based devices.

**Table 2:** Summary of energy loss data (unit: eV) for 0%, 35%, and 100% blend‐based devices

IEICO‐4F ratios	*E*_gap_	Measured *qV*_oc_	$qV_{\mathrm{oc}}^{\mathrm{Calc}}$	Δ*E*	$qV_{\mathrm{oc}}^{\mathrm{SQ}}$	$qV_{\mathrm{oc}}^{\mathrm{rad}}$	Δ*E*_1_	Δ*E*_2_	Δ*E*_3_
							$E_{\mathrm{gap}}\;-\;qV_{\mathrm{oc}}^{\mathrm{SQ}}$	$q\Delta V_{\mathrm{oc}}^{\mathrm{red},\mathrm{below}\;\mathrm{gap}}$	$q\Delta V_{\mathrm{oc}}^{\mathrm{non‐rad}}$
0%	1.60	0.80	0.80	0.80	1.31	1.18	0.29	0.13	0.38
35%	1.38	0.78	0.78	0.59	1.10	1.06	0.27	0.04	0.28
100%	1.35	0.75	0.75	0.60	1.07	1.04	0.28	0.04	0.28

In summary, a type of TOSC with a significant current enhancement of 44% and a small energy loss of 0.59 eV is successfully demonstrated by introducing a near‐infrared nonfullerene acceptor (IEICO‐4F) as the third component to the binary blend of PBDB‐T:HF‐PCIC. Comparing with binary PBDB‐T:HF‐PCIC solar cells, ternary PBDB‐T:HF‐PCIC:IEICO‐4F solar cells exhibit enhanced *J*_sc_ from 16.26 to 23.46 mA cm^−2^, and reduced energy loss from 0.80 to 0.59 eV. As a result, PCE is increased from 8.82% of binary OSC to 11.20% of ternary OSC. It is proved that the third component IEICO‐4F can not only broaden the absorption range but also work as morphology modifier in our TOSC, and there also exists efficient energy transfer between HF‐PCIC and IEICO‐4F. These reasons should account for the greatly enhanced *J*_sc_. Furthermore, the reducing in both voltage losses caused by radiative recombination from the absorption below the band gap and nonradiative recombination after introducing IEICO‐4F is the reason for the small energy loss of 0.59 eV and a high *V*_oc_ of 0.78 eV for TOSCs. Our results indicate that combining two nonfullerene acceptors with complementary absorption and divergent crystallinity is effective to achieve a significant current improvement via the realization of energy transfer and optimized morphology. Besides, the component with the narrowest band gap in the ternary blend had better be a low energy loss material, so as to reduce the impact of extending absorption range on the *V*_oc_. Thus, our designing strategy can provide good guidelines for the future development of TOSCs.

## Conflict of Interest

The authors declare no conflict of interest.

## Supporting information

Supplementary
