# Supplementary material for: A Near‐Infrared Photoactive Morphology Modifier Leads to Significant Current Improvement and Energy Loss Mitigation for Ternary Organic Solar Cells
Source: Adv Sci (Weinh). 2018 Jun 20;5(8):1800755. doi: 10.1002/advs.201800755 (PMC6097004; doi:10.1002/advs.201800755)
Supplement: Supplementary file 1 — Supplementary [file ADVS-5-1800755-s001.pdf]

Copyright WILEY-VCH Verlag GmbH & Co. KGaA, 69469 Weinheim, Germany, 2018.

ADVANCED SCIENCE
Open Access

Supporting Information

for *Adv. Sci.,* DOI: 10.1002/advs.201800755

A Near-Infrared Photoactive Morphology Modifier Leads to Significant Current Improvement and Energy Loss Mitigation for Ternary Organic Solar Cells

Lingling Zhan, Shuixing Li, Huotian Zhang, Feng Gao, Tsz-Ki Lau, Xinhui Lu,* Danyang Sun, Peng Wang, Minmin Shi,* Chang-Zhi Li, and Hongzheng Chen**

Copyright WILEY-VCH Verlag GmbH & Co. KGaA, 69469 Weinheim, Germany, 2018.

Supporting Information

A Near-Infrared Photoactive Morphology Modifier Leads to Significant Current Improvement and Energy Loss Mitigation for Ternary Organic Solar Cells

Lingling Zhan, Shuixing Li, Huotian Zhang, Feng Gao, Tsz-Ki Lau, Xinhui Lu,* Danyang Sun, Peng Wang, Minmin Shi*, Chang-Zhi Li, and Hongzheng Chen**

L. Zhan and S. Li contributed equally to this work.

L. Zhan, Dr. S. Li, Prof. M. Shi, Prof. C.-Z. Li, Prof. H. Chen
State Key Laboratory of Silicon Materials, MOE Key Laboratory of Macromolecular Synthesis and Functionalization, Department of Polymer Science and Engineering, Zhejiang University, Hangzhou 310027, P. R. China
E-mail: minminshi@zju.edu.cn; hzchen@zju.edu.cn
H. Zhang, Prof. F. Gao
Biomolecular and Organic Electronics, IFM, Linköping University, Linköping 58183, Sweden
E-mail: fenga@ifm.liu.se
T.-K. Lau, Prof. X. Lu
Department of Physics, Chinese University of Hong Kong, New Territories, Hong Kong, P. R. China
E-mail: xhlu@phy.cuhk.edu.hk
D. Sun, Prof. P. Wang
Department of Chemistry, Zhejiang University, Hangzhou 310027, P. R. China

Materials and Methods

Instrument. ^{1}H NMR, ^{13}C NMR and ^{19}F NMR spectra were obtained on an Agilent 600 MHz DD2 nuclear magnetic resonance (NMR) spectroscope. MALDI-TOF MS spectra were measured on a Walters Maldi Q-TOF Premier mass spectrometry. UV-vis absorption spectra were recorded on a Shimadzu UV-2450 spectrophotometer. Thermogravimetric analysis (TGA) was carried out on a WCT-2 thermal balance under protection of nitrogen at a heating rate of 10 ^{o}C/min. Differential scanning calorimetry (DSC) was recorded on a Pekin-Elmer Pyris 1 differential scanning calorimeter. Cyclic voltammetry (CV) was done on a CHI600A electrochemical workstation with Pt disk, Pt plate, and standard calomel electrode (SCE) as working electrode, counter electrode, and reference electrode, respectively, in a 0.1 mol/L tetrabutylammoniumhexafluorophosphate (Bu_4NPF_6) CH_2Cl_2 solution. The CV curves were recorded versus the potential of SCE, which was calibrated by the ferrocene-ferrocenium (Fc/Fc^+) redox couple (4.8 eV below the vacuum level). Steady state Fluorescence spectra were measured by HORIBA Jobin Yvon instrument Fluorolog at room temperature. Topographic images of the films were obtained on a VeecoMultiMode atomic force microscopy (AFM) in the tapping mode using an etched silicon cantilever at a nominal load of ~2 nN, and the scanning rate for a 10 μm×10 μm image size was 1.5 Hz. Transmission electron microscopy (TEM) images were performed on JEOL-1010 at 80 KV accelerating voltage in bright field mode.

Materials. All reagents and solvents, unless otherwise specified, were purchased from J&K Scientific, Derthon Tech, Suna Tech, Aldrich and Energy Chemical Ltd. and were used without further purification. **PBDB-T** and **IEICO-4F** were purchased from Solarmer Materials Inc.

DFT Calculation. Geometry optimizations were carried out by the density functional theory (DFT) method at the B3LYP/6-31G level. All the calculations were performed using Gaussian 03 program. All ethylhexyl substituents were replaced with methyl groups in calculations.

Transient Absorption Spectroscopy Measurements. Briefly, a portion of the output from a 1 kHz Coherent Astrella Ti:Sapphire amplifier (7 mJ, 35 fs at 800 nm) was split into the pump and probe beams. The pump beam was directed into an optical parametric amplifier (Coherent OPerA Solo) to generate tunable excitation (490 nm) while the probe beam was passed through an optical delay line and then focused on a sapphire to afford a supercontinuum white light. The white light was split into two almost-equal beams as the probe and reference lights. The pump and probe beams were focused and overlapped into a spot on the sample, with the relative polarizations of the pump and probe beams set at the magic angle. The transient spectra and kinetics were obtained using a Helios Fire transient absorption spectrometer (Ultrafast Systems), averaging at least three scans and using 3 s of averaging at every given time delay.

GIWAXS Measurements. GIWAXS measurements were carried out with a Xeuss 2.0 SAXS/WAXS laboratory beamline using a Cu X-ray source (8.05 keV, 1.54 Å) and a Pilatus3R 300K detector. The incidence angle is 0.2^{o}.

FTPS-EQE Measurements. FTPS-EQE was measured using Vertex 70 from Bruker Optics, equipped with a quartz tungsten halogen lamp, quartz beam splitter and external deterctor option. A low-noise current amplifer (SR570) was used to amplify the photocurrent produced on illumination of the photovoltaic devices with light modulated by the Fourier transform infrared spectroscope (FTIR). The output voltage of the current amplifier was fed back into

the external detector port of the FTIR, to be able to use the FTIR's software to collect the phto current spectrum.

EL Measurements. EL spectra were measured using a light guide positioned close to the sample. The bias was applied on the devices using a Keithley 2400 SourceMeter. The detector was a Newton EM-CCD Si array detector at -60 ºC with a Shamrock SR-303i spectrography from Andor Tech. EQE_{EL} values were obtained from an in-house-built system including a Hamamatsu silicon photodiode 1010B, a Keithley 2400 SourceMeter to provide voltage and record injected current, and a Keithley 485 Picoammeter to measure the emitted light intensity.

Device Fabrication and Characterization. Organic solar cells were fabricated on glass substrates commercially pre-coated with a layer of indium tin oxide (ITO) with the inverted structure of ITO/ZnO/PFN/Active Layer/MoO_3/Ag. Prior to fabrication, the substrates were cleaned using detergent, deionized water, acetone and isopropanol consecutively for every 15 min, and then treated in an ultraviolet ozone generator for 15 min. A thin layer (~30 nm) of ZnO was spin coated onto precleaned ITO-coated glass substrates at 3500 rpm for 60 s and then annealed at 170 ^{o}C for 20 min. Then the substrates were transferred to a glovebox, and a thin layer (~5 nm) of PFN was spin coated on ZnO at 3000 rpm for 60 s from 0.4 mg/mL solution. After that, the active layer was spin coated from 20 mg/mL chlorobenzene solution (D:A=1:1.2, 0.8% CN) at 2100 rpm for 60 s to form an active layer of around 100 nm. For the devices needed annealing, an extra pre-annealing at 110 ^{o}C or other temperatures for 10 min was performed. Finally, a layer of MoO_3 (10 nm) and the Ag (100 nm) electrode were deposited by thermal evaporation to complete the device with an active area of 6 mm^2.

The current density-voltage (*J-V*) curves of OSCs were measured with Keithley 236 measurement source units under 1 sun, AM 1.5 G spectra from a solar simulator (Taiwan,

Enlitech), and the light intensity was calibrated with a standard photovoltaic (PV) reference cell. The external quantum efficiency (EQE) spectra were measured with a Stanford lock-in amplifier 8300 unit.

The charge carrier mobilities of the binary and ternary blended films were measured using the space-charge-limited current (SCLC) method. Hole-only devices were fabricated in a structure of ITO/PEDOT:PSS/Active Layer/MoO_3/Ag, electron-only devices were fabricated in a structure of ITO/ZnO/PFN/Active Layer/PFN/Al. The device characteristics were extracted by modeling the dark current under forward bias using the SCLC expression described by the Mott-Gurney law:

$$J = \frac{9}{8}\varepsilon_r \varepsilon_0 \mu \frac{V^2}{L^3} \quad (1)$$

Here, $\varepsilon_r \approx 3$ is the average dielectric constant of the blend film, ε_0 is the permittivity of the free space, μ is the carrier mobility, $L \approx 100$ nm is the thickness of the film, and V is the applied voltage.

Synthesis of HF-PCIC

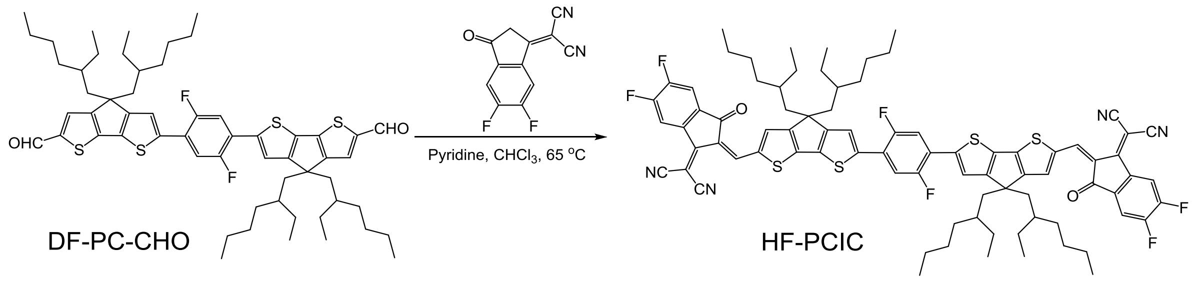

2,2'-((2Z,2'Z)-(((2,5-difluoro-1,4-phenylene)bis(4,4-bis(2-ethylhexyl)-4H-cyclopenta[2,1-b:3,4-b']dithiophene-6,2-diyl))bis(methanylylidene))bis(5,6-difluoro-3-oxo-2,3-dihydro-1H-indene-2,1-diylidene))dimalononitrile (HF-PCIC).

To a two-necked round bottom flask were added DF-PC-CHO (0.3g, 0.3 mmol), 2-(5,6-difluoro-3-oxo-2,3-dihydro-1H-inden-1-ylidene)malononitrile (0.35 g, 1.5 mmol) and dried $CHCl_3$ (50 mL). The mixture was frozen with liquid nitrogen, followed by three times of successive vacuum and nitrogen fill cycles, then under the protection of nitrogen, 0.6 mL pyridine was added. The resulting mixture was refluxed at 65 ^{o}C for 12 h. After removing the solvent, the residue was purified using silica gel column chromatography with hexane/dichloromethane (1:3) as the eluent, yielding a deep brown solid (0.27 g, 64.5%). ^{1}H NMR (600 MHz, $CDCl_3$): δ = 8.91 (s, 2H), 8.53 (dd, *J* = 9.8, 6.5 Hz, 2H), 7.68 (t, *J* = 7.3 Hz, 4H), 7.54-7.50 (m, 2H), 7.48 (t, *J* = 6.6 Hz, 2H), 2.07-1.94 (m, 8H), 1.09-0.89 (m, 32H), 0.79-0.68 (m, 16H), 0.64 (t, *J* = 7.3 Hz, 12H). ^{13}C NMR (600 MHz, $CDCl_3$): δ = 186.08, 165.70, 159.75, 158.41, 157.66, 155.93, 155.15, 154.28, 153.50, 153.41, 143.06, 139.29, 138.25, 137.97, 136.52, 134.46, 122.65, 119.91, 114.98, 114.84, 114.54, 112.55, 112.42, 107.35, 68.53, 54.27, 43.24, 43.13, 35.55, 35.50, 34.31, 34.05, 28.50, 27.53, 27.29, 22.79, 22.78, 14.06, 13.98, 10.61, 10.59. ^{19}F NMR (600 MHz, $CDCl_3$): δ = -116.86, -123.37, -124.48. MS (MALDI-TOF): Calcd for $C_{82}H_{80}F_6N_4O_2S_4$ (M^+): 1395.80, Found: 1396.57.

NMR Spectra

^{1}H NMR Spectrum

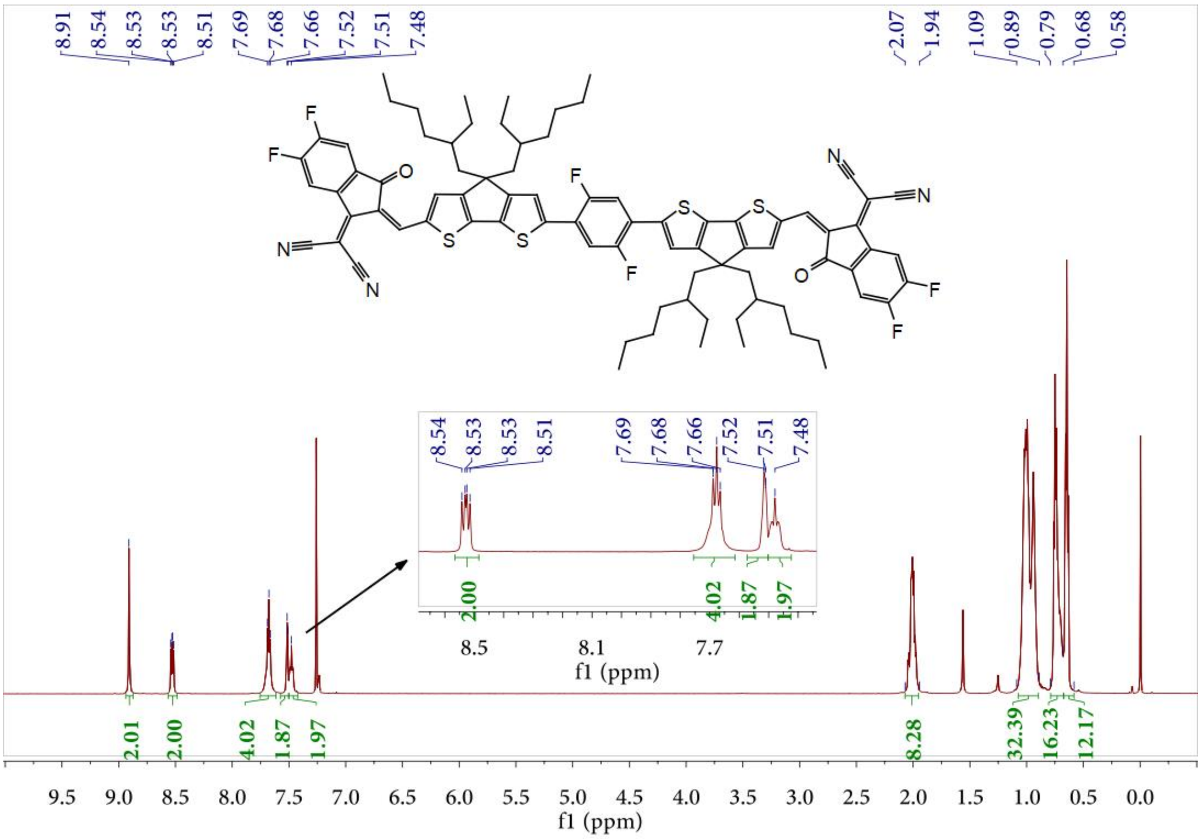

^{13}C NMR Spectrum

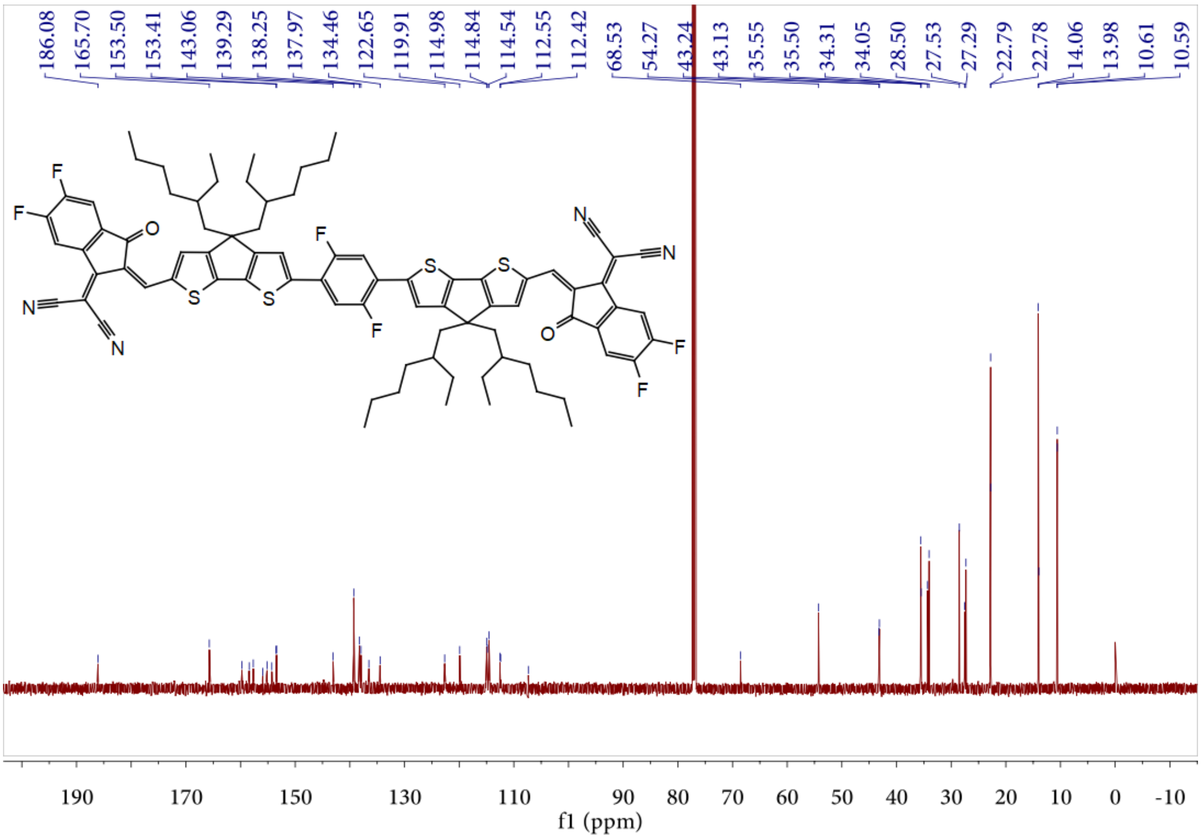

^{19}F NMR Spectrum

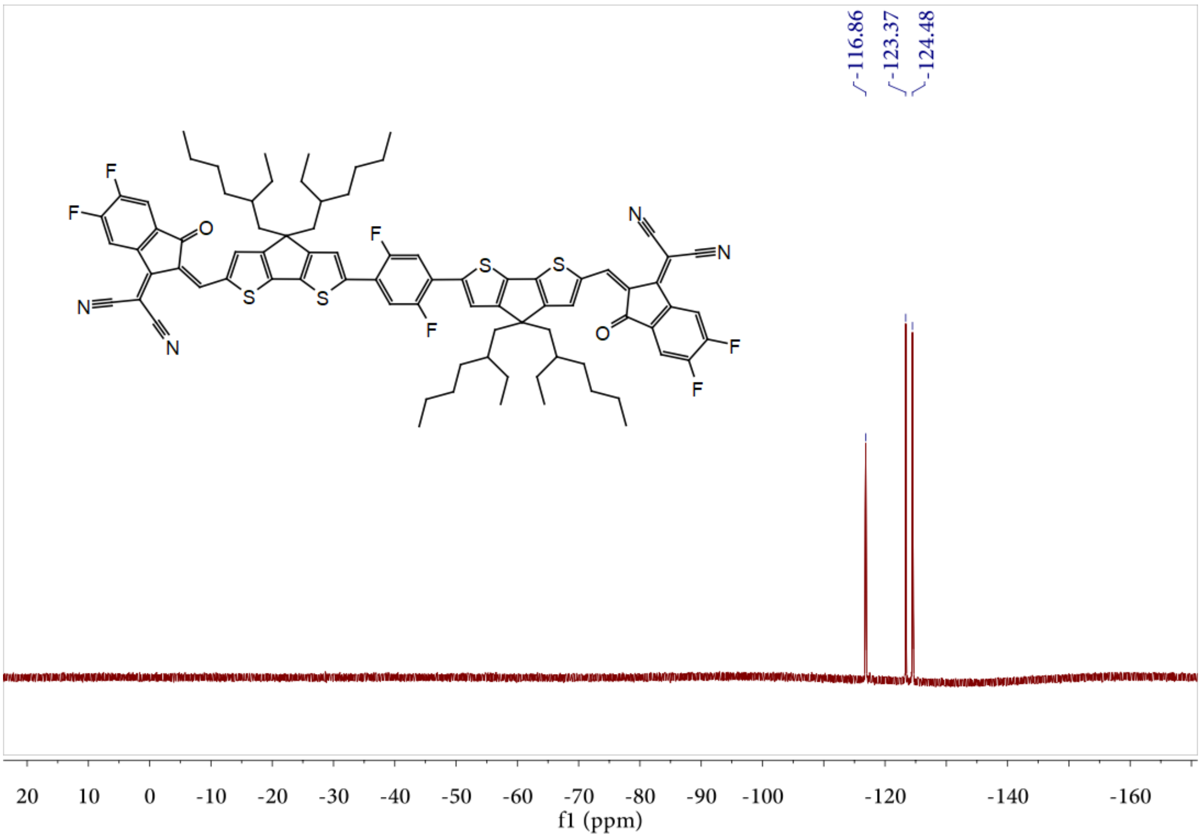

Supporting Figures

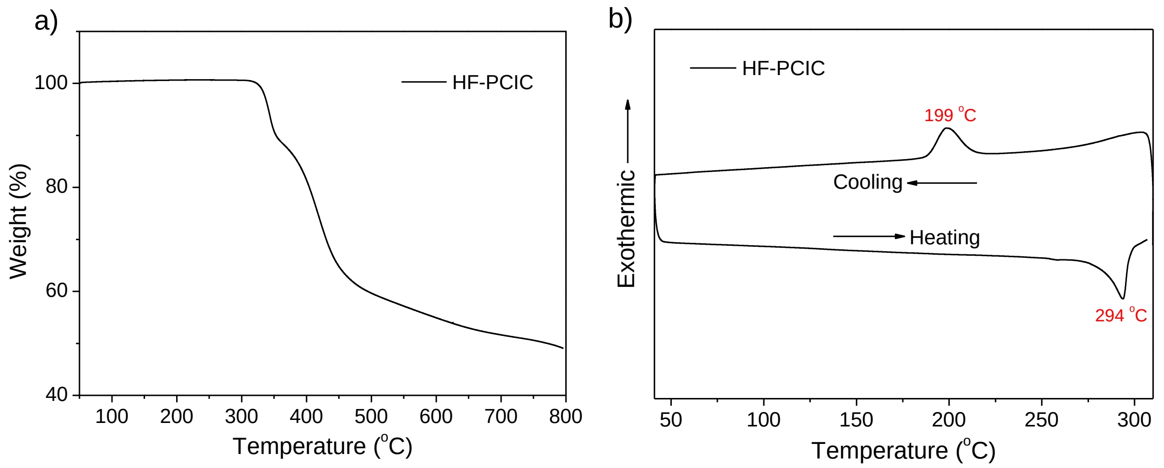

Figure S1. a) TGA curve of HF-PCIC. b) DSC curve of HF-PCIC.

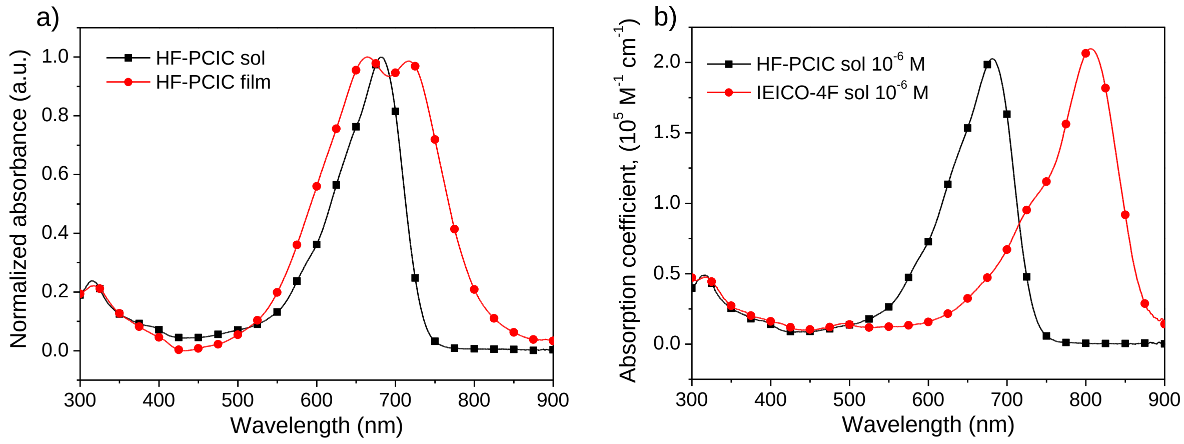

Figure S2. a) UV-vis absorption spectra of HF-PCIC in chloroform solution and thin film. b) UV-vis absorption spectra of HF-PCIC and IEICO-4F in chloroform solutions with 10^{-6} M concertrations.

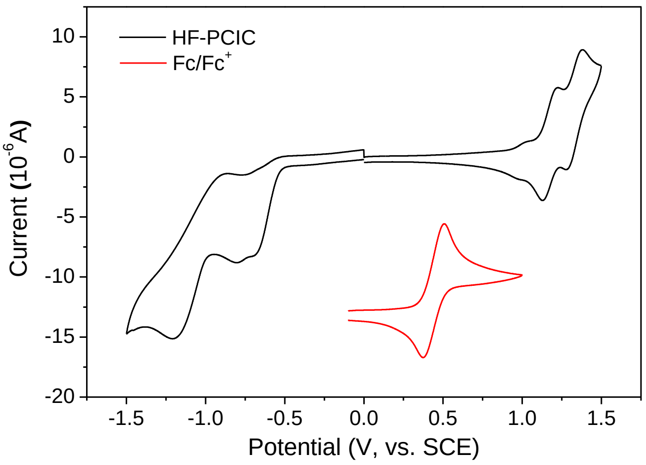

Figure S3. Cyclic voltammograms of HF-PCIC and Fc/Fc^+ in dichloromethane solutions.

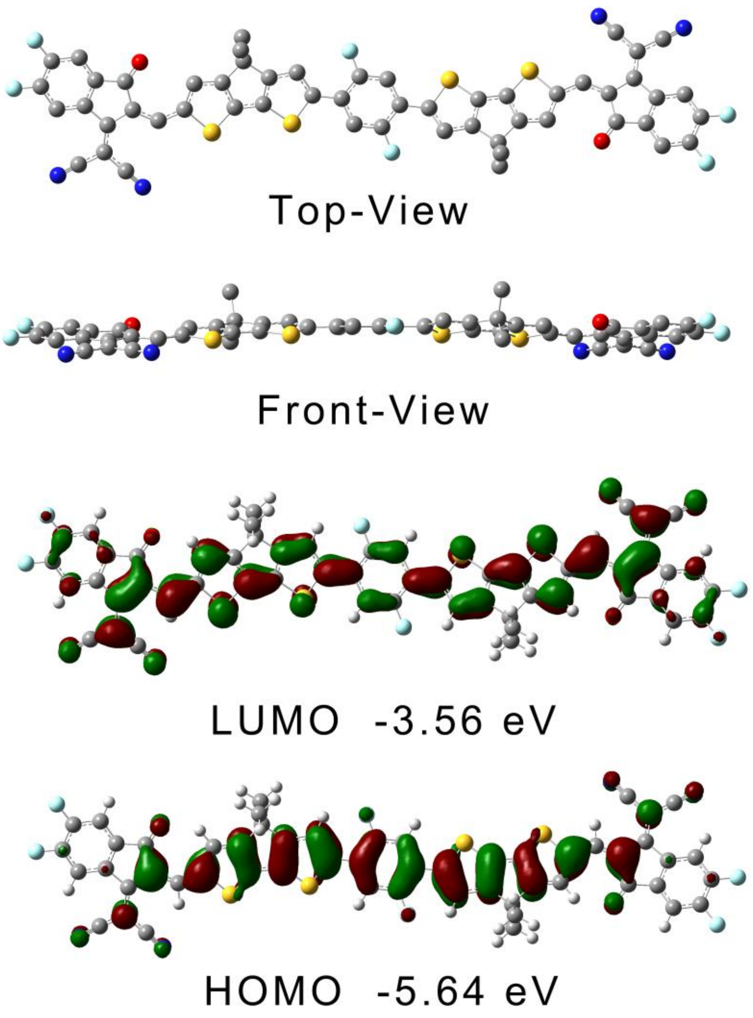

Figure S4. Simulated molecular geometries (Top-View and Front-View) and frontier molecular orbitals by DFT calculation for HF-PCIC.

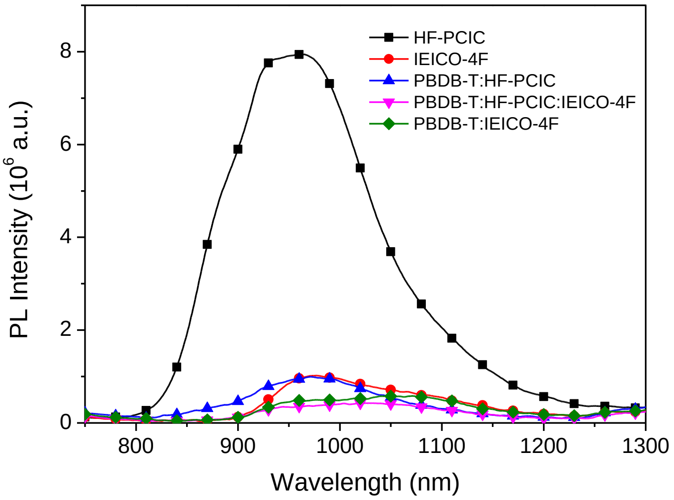

Figure S5. Photoluminescence spectra of HF-PCIC, IEICO-4F, two binary blends and one ternary blend excited at 680 nm.

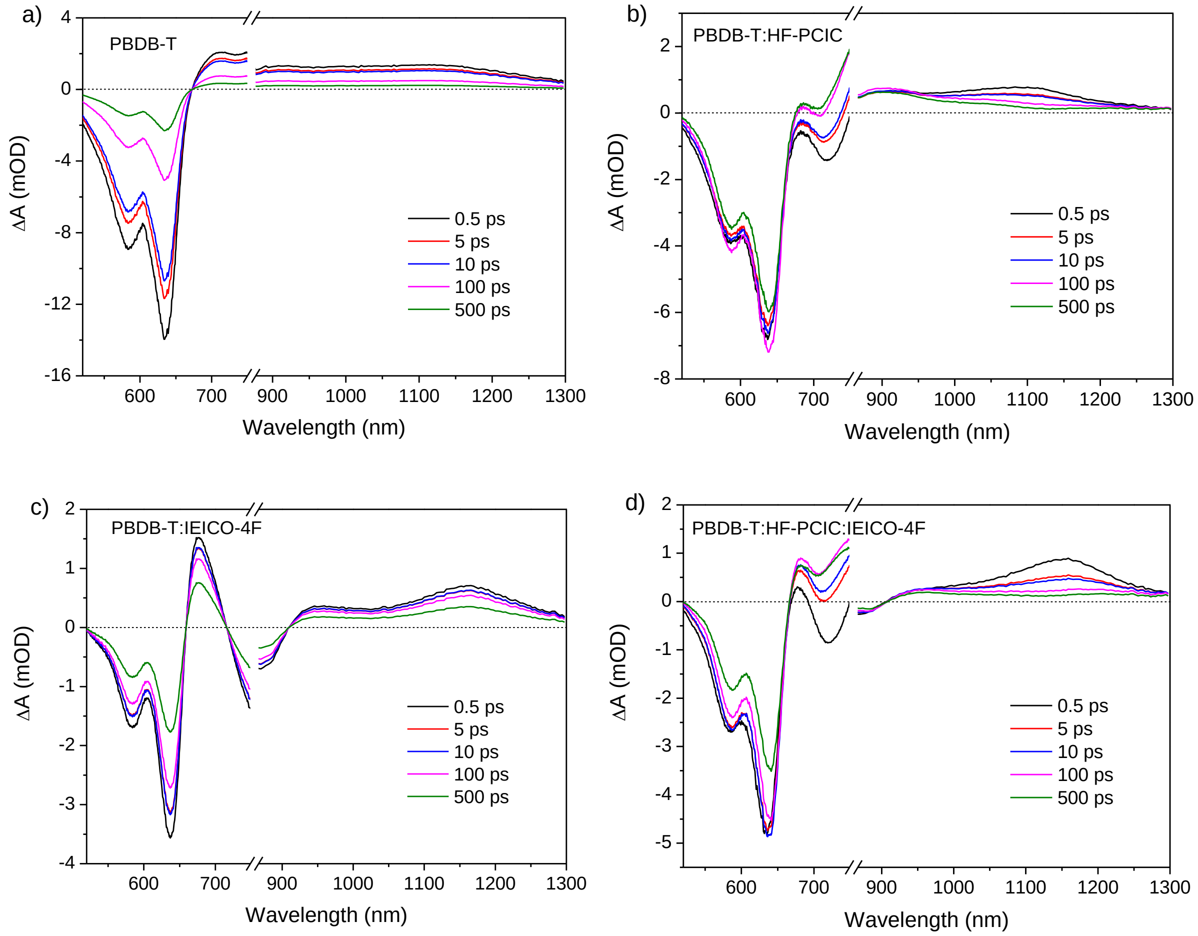

Figure S6. Transient absorption spectra of a) neat PBDB-T film, b) blended PBDB-T:HF-PCIC film, c) blended PBDB-T:IEICO-4F film and d) ternary blend with 35% IEICO-4F.

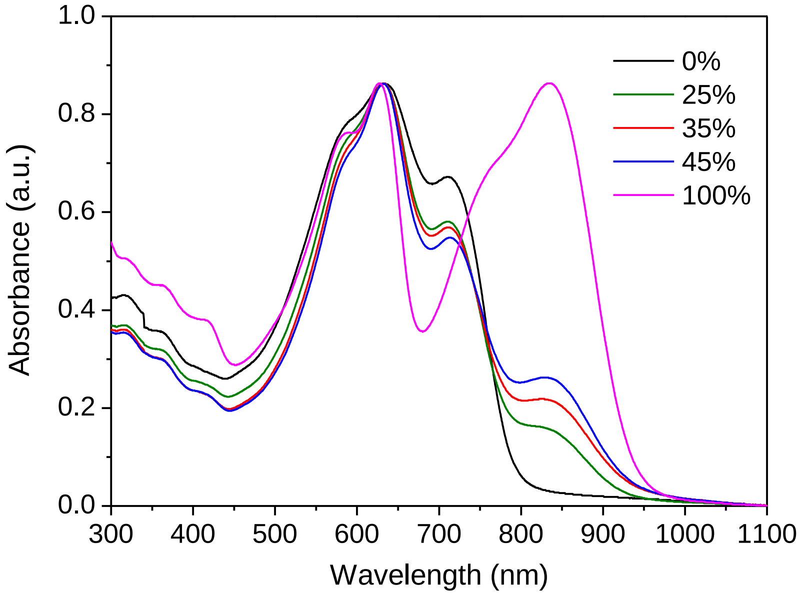

Figure S7. UV-vis absorption spectra of the donor/acceptor blends with different weight ratios of IEICO-4F.

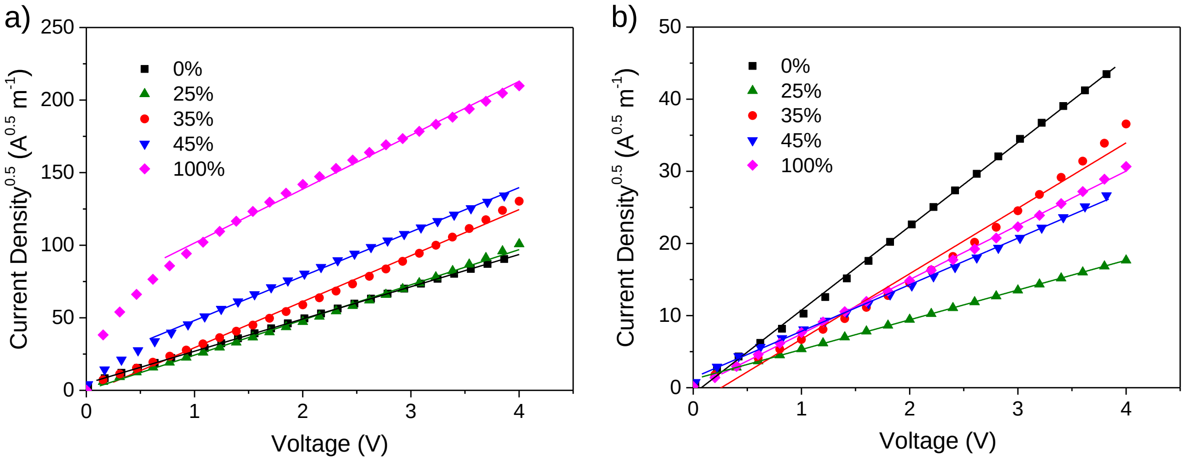

Figure S8. a) $J^{0.5}$-V curves of the hole-only devices based on PBDB-T:HF-PCIC:IEICO-4F films with different weight ratios of IEICO-4F. b) $J^{0.5}$-V curves of the electron-only devices based on PBDB-T:HF-PCIC:IEICO-4F films with different weight ratios of IEICO-4F.

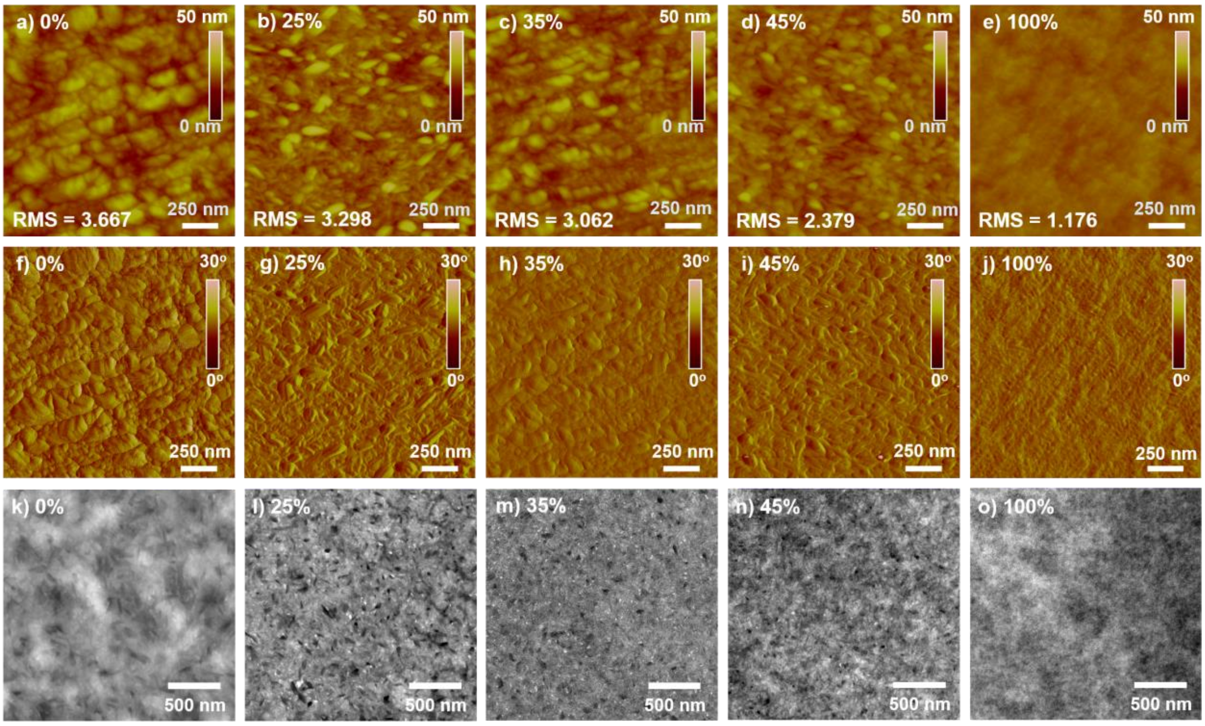

Figure S9. a-e) AFM height images, f-j) AFM phase images and k-o) TEM images of the donor/acceptor blends with different weight ratios of IEICO-4F.

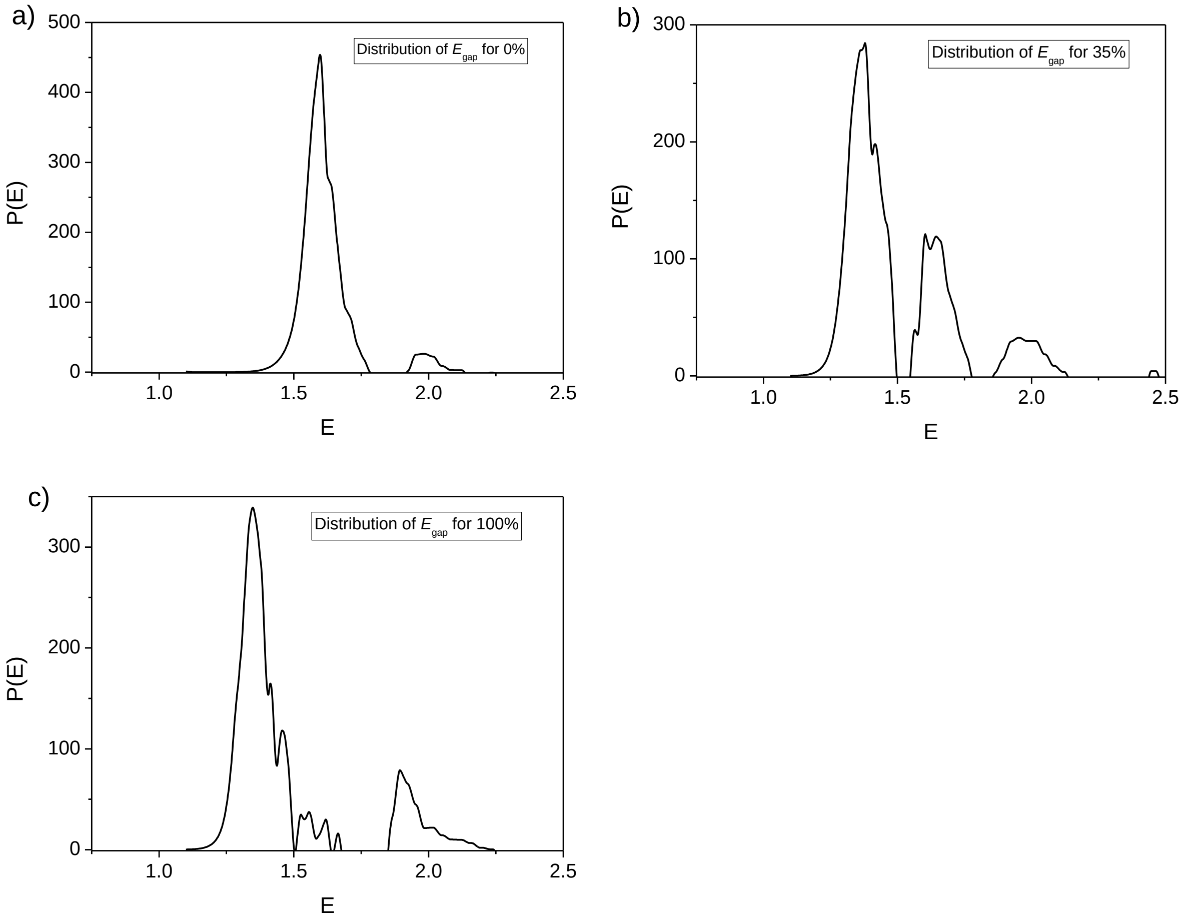

Figure S10. Distributions of E_{gap} for a) 0%, b) 35% and c) 100% blends using the derivatives of EQEs.

Supporting Notes

Note 1

The V_{oc} of any type of solar cells is determined by the ratio between short circuit current (J_{sc}) and dark saturation current (J_0), following this expression:

$$V_{oc} = \frac{kT}{q}\ln\left(\frac{J_{sc}}{J_0} + 1\right) \qquad (2)$$

Where k is the boltzmann constant, T is the temperature, and q is the elementary charge. The expression for J_{sc} and J_0 are given by:

$$J_{sc} = q \cdot \int_0^{\infty} EQE_{PV}(E) \cdot \emptyset_{AM1.5}(E)dE \qquad (3)$$

$$J_0 = \frac{q}{EQE_{EL}} \cdot \int_0^{\infty} EQE_{PV}(E) \cdot \emptyset_{BB}(E)dE \qquad (4)$$

The expression for J_0 is the Rau's reciprocity relation, where EQE_{EL} is radiative quantum efficiency of the solar cell when charge carriers are injected into the device in dark, and ϕ_{BB} is the black body spectrum.

When all the recombination is radiative (i.e. EQE_{EL} = 1), J_0 is minimized, and V_{oc} is maximized:

$$J_0^{rad} = q \cdot \int_0^{\infty} EQE_{PV}(E) \cdot \emptyset_{BB}(E)dE \qquad (5)$$

$$V_{oc}^{rad} = \frac{kT}{q}\ln\left(\frac{J_{sc}}{J_0^{rad}} + 1\right) = \frac{kT}{q}\ln(\frac{q \cdot \int_0^{\infty} EQE_{PV}(E) \cdot \emptyset_{AM1.5}(E)dE}{q \cdot \int_0^{\infty} EQE_{PV}(E) \cdot \emptyset_{BB}(E)dE} + 1) \qquad (6)$$

In the Shockley-Queisser theory, the general quantum efficiency $EQE_{PV}^{SQ}(E)$ can be defined as follow:

$$EQE_{PV}^{SQ}(E) = 1, \quad E \geq E_{gap}\,; \qquad EQE_{PV}^{SQ}(E) = 0, \quad E < E_{gap} \qquad (7)$$

Substituting general quantum efficiency $EQE_{PV}^{SQ}(E)$ (equation 7) in equation 5, then we can get the saturation current in the SQ limit, J_0^{SQ}.

$$J_0^{SQ} = q \cdot \int_{E_{gap}}^{\infty} EQE_{PV}^{SQ}(E) \cdot \emptyset_{BB}(E)dE = q \cdot \int_{E_{gap}}^{\infty} \emptyset_{BB}(E)dE \qquad (8)$$

In the same way, we can calculate the value of the SQ open-circuit voltage limit, V_{oc}^{SQ}, according to equation 6,

$$V_{oc}^{SQ} = \frac{kT}{q}\ln\left(\frac{J_{sc}}{J_0^{SQ}} + 1\right) = \frac{kT}{q}\ln\left(\frac{q \cdot \int_0^{\infty} EQE_{PV}(E) \cdot \emptyset_{AM1.5}(E)dE}{q \cdot \int_0^{\infty} \emptyset_{BB}(E)dE} + 1\right) \qquad (9)$$

The difference between V_{oc}^{SQ} and V_{oc}^{rad} is due to that in the SQ theory, the band edge of the absorber is totally abrupt when calculating V_{oc}^{rad}, the band gap will be smeared out for the existence of charge transfer state absorption.

Therefore, we can deduce the voltage loss of radiative recombination below the gap, $V_{oc}^{rad,below\ gap}$.

$$V_{oc}^{rad,below\ gap} = V_{oc}^{SQ} - V_{oc}^{rad} \qquad (10)$$

The voltage loss due to non-radiative recombination, $V_{oc}^{non-rad}$, can be rewritten as

$$V_{oc}^{non-rad} = V_{oc}^{rad} - V_{oc} = -\frac{kT}{q}\ln(EQE_{EL}) \qquad (11)$$

Based on the previous disscussions, we are now able to summarize the energy loss from the E_{gap} to the qV_{oc} for any type of solar cells.

$$q\Delta \mathrm{V} = \Delta E_1 + \Delta E_2 + \Delta E_3$$

$$= \left(E_{gap} - qV_{oc}^{SQ}\right) + q\Delta V_{oc}^{rad,below\ gap} + q\Delta V_{oc}^{non-rad} \qquad (12)$$

Therefore, we can get these three terms of energy losses based on related experiments and calculations.

Note 2

In most literatures, the E_{gap} is identified by the absorption edge (E_{gap}^{opt}) of the narrowest materials, however, the real E_{gap} may vary after mixing the donor and acceptor and post-treatment. A more accurate method to obtain E_{gap} is proposed by Uwe Rau, which we use here. In this method, we firstly use the derivative of EQE edges to get the distributions of E_{gap} (Figure S10):

$$\frac{d}{dE}Q_e^{PV}(E) = \int_{-\infty}^{\infty} P(E_{gap})\frac{d}{dE}H(E-E_g)dE_g = \int_{-\infty}^{\infty} P(E_{gap})\delta(E-E_g)dE_g = P(E) \qquad (13)$$

Then we define average E_{gap} through integration:

$$E_{gap}^{PV} = \int_a^b E_{gap}P(E_{gap})dE_{gap} / \int_a^b P(E_{gap})dE_g \qquad (14)$$

Note 3

The value of EQE_{EL} is obtained through the following method: We apply a current to the device and measure its luminous efficiency, when the current equals to the short-circuit current of the device, the measured luminous efficiency is the value of EQE_{EL}.

Supporting Tables

Table S1. Photovoltaic parameters of the binary OSCs based on PBDB-T:HF-PCIC blended films under the illumination of AM 1.5 G, 100 mWcm^{-2}

D/A (w/w)	CN (%)	Annealing Temp. (°C)[a]	V_{oc} (V)	J_{sc} (mA cm^{-2})	FF (%)	PCE (%)
1:1	w/o	w/o	0.80	13.92	44.49	5.07
1:1.2	w/o	w/o	0.78	14.00	47.66	5.34
1:1.5	w/o	w/o	0.77	13.82	46.01	5.03
1:1.2	0.5	w/o	0.79	15.59	64.01	7.81
1:1.2	0.8	w/o	0.79	15.20	64.54	7.93
1:1.2	1.0	w/o	0.75	14.67	64.54	7.00
1:1.2	0.8	100	0.79	15.79	67.35	8.38
1:1.2	0.8	110	0.80	16.26	68.44	8.82
1:1.2	0.8	120	0.78	15.90	67.68	8.61

[a] Annealing for 10 min.

Table S2. Photovoltaic parameters of the ternary OSCs with different weight ratios of IEICO-4F under the illumination of AM 1.5 G, 100 mWcm^{-2}

IEICO-4F ratios	CN (%)	Annealing Temp. (°C)[a]	V_{oc} (V)	J_{sc} (mA cm^{-2})	FF (%)	PCE (%)
25%	0.8	110	0.79	20.34	63.01	10.41
35%	0.8	110	0.78	23.46	60.99	11.20
45%	0.8	110	0.78	20.71	60.02	9.93
55%	0.8	110	0.76	19.49	50.79	7.59
65%	0.8	110	0.76	20.05	45.71	7.01
75%	0.8	110	0.76	18.68	45.85	6.50
85%	0.8	110	0.76	17.74	43.18	5.82

Table S3. The lifetime constants fitting from transient absorption spectra.

Sample	τ_1 / ps	τ_2 / ps	τ_3 / ps	τ_4 / ps
HF-PCIC	0.14	18.76	102.73	480.86
IEICO-4F	0.18	3.18	42.19	698.21
HF-PCIC:IEICO-4F	0.15	1.95	58.91	3205.94

Table S4. Electron and hole mobilities of PBDB-T:HF-PCIC:IEICO-4F films with different weight ratios of IEICO-4F

IEICO-4F ratios[a)]	μ_h (× 10^{-4} cm^2 V^{-1} s^{-1})	μ_e (× 10^{-4} cm^2 V^{-1} s^{-1})	μ_h / μ_e
0%	1.63 ± 0.29	0.47 ± 0.01	3.5
25%	2.21 ± 0.26	0.06 ± 0.01	36.8
35%	3.31 ± 0.24	0.32 ± 0.08	10.3
45%	3.01 ± 0.17	0.15 ± 0.01	20.1
100%	3.88 ± 1.39	0.19 ± 0.02	20.4

[a)] The weight ratios of IEICO-4F in the acceptor mixture, and the total D/A weight ratio is fixed as 1:1.2.

Table S5. Summary of photovoltaic parameters, current enhancement and energy losses of ternary solar cells

Blend	V_{oc} (V)	J_{sc} (mA cm^{-2})	FF	PCE (%)	ΔJ_{sc} (mA cm^{-2})$^{a)}$	Ref.
PBDB-T/HF-PCIC/IEICO-4F	0.78	23.46	0.61	11.20	7.20	This Work
PTB7-Th/PBDB-T/SFBRCN	0.93	17.86	0.74	12.27	0.82	[1]
PBDB-T/ITCN/IT-M	0.95	17.87	0.71	12.16	0.62	[2]
PTB7-Th/IHIC/IHIC-N	0.78	21.30	0.71	11.90	1.90	[3]
PBDB-T/PTB7-Th/IEICO-4F	0.74	24.14	0.65	11.62	1.29	[4]
J52/IT-M/IEICO	0.85	19.70	0.67	11.10	2.60	[5]
PSTZ/ITIC/IDIC	0.95	17.40	0.67	11.10	2.70	[6]
J52/PTB7-Th/IEICO-4F	0.73	25.30	0.59	10.90	2.50	[7]
PDBT-T1/Sdi-PBI-Se/ITIC-Th	0.93	15.37	0.70	10.10	2.73	[8]
PTB7-Th/IDT-2BR/PDI-2DTT	1.03	14.50	0.65	9.70	1.70	[9]
J51/PTB7-Th/ITIC	0.81	17.75	0.68	9.70	1.56	[10]
PTB7-Th/PBDTTS-FTAZ/PNDI-T10	0.84	14.40	0.74	9.00	1.50	[11]
P3HT/IDTBR/IDFBR	0.82	14.40	0.64	7.70	0.50	[12]
PTB7-Th/PCDTBT/ITIC	0.80	16.71	0.56	7.51	2.82	[13]
PTB7-Th/PBDD-ff4T/N2200	0.82	15.70	0.56	7.20	1.80	[14]
PBDB-T/IT-M/Bis[70]PCBM	0.95	17.39	0.74	12.20	0.69	[15]
PTB7-Th/DR3TSBDT/$PC_{71}BM$	0.77	23.31	0.70	12.10	3.61	[16]
PDOT/ITIC/$PC_{71}BM$	0.96	17.49	0.67	11.21	3.57	[17]
PTB7-Th/PDT2FBT-ID/$PC_{71}BM$	0.77	18.92	0.76	11.10	1.02	[18]
PTB7/DPPEZnP-THE/$PC_{71}BM$	0.77	18.68	0.75	10.79	1.72	[19]
p-DTS($FBTTh_2$)$_2$/ZnP/$PC_{71}BM$	0.79	17.64	0.76	10.62	3.11	[20]
PTB7-Th/PBTZT-STAT-BDTT-8/$PC_{71}BM$	0.77	18.67	0.71	10.21	1.99	[21]
PTB7-Th/PffBT4T-2OD/$PC_{71}BM$	0.78	19.02	0.73	10.72	1.23	[22]
DRTB-T/IDIC/$PC_{71}BM$	0.99	15.47	0.68	10.48	1.16	[23]
PTB7-Th/BTR/$PC_{71}BM$	0.78	17.10	0.75	10.10	0.30	[24]
PTB7-Th/*p*-DTS($FBTTh_2$)$_2$/$PC_{71}BM$	0.76	18.44	0.75	10.50	0.91	[25]
PPBDTBT/ITIC/$PC_{71}BM$	0.89	16.66	0.68	10.35	3.66	[26]
DRCN5T/BTR/$PC_{71}BM$	0.90	16.54	0.68	10.05	0.77	[27]
DRCN5T/DR3TSBDT/$PC_{71}BM$	0.92	16.50	0.67	10.16	0.90	[28]
PDBT-T1/ITIC-Th/$PC_{71}BM$	0.93	15.54	0.71	10.22	2.30	[29]
PTB7-Th/TPE-4PDI/$PC_{71}BM$	0.78	17.44	0.74	10.09	0.97	[30]
PTB7-Th/PDBT-T1/$PC_{71}BM$	0.81	17.80	0.70	9.90	1.70	[31]
PTB7-Th/PID2/$PC_{71}BM$	0.78	16.68	0.71	9.20	1.76	[32]
PPDT2FBT/PPDT2CNBT/$PC_{71}BM$	0.77	18.10	0.66	9.21	1.30	[33]
PTB7/PCDTBT/$PC_{71}BM$	0.80	17.10	0.65	8.90	2.40	[34]
PTB7/Si-PCPDTBT/$PC_{71}BM$	0.70	15.94	0.77	8.60	0.93	[35]
PTB7/PID2/$PC_{71}BM$	0.72	16.80	0.69	8.22	1.80	[36]
PBTA-BO/IFBR/$PC_{61}BM$	0.93	13.45	0.65	8.11	4.52	[37]

$^{a)}$ Current enhancement from the binary (higher efficiency or more content) to ternary solar cells.

[1] X. Xu, Z. Bi, W. Ma, Z. Wang, W. C. H. Choy, W. Wu, G. Zhang, Y. Li, Q. Peng, *Adv. Mater.* **2017**, *29*, 1704271.
[2] W. Jiang, R. Yu, Z. Liu, R. Peng, D. Mi, L. Hong, Q. Wei, J. Hou, Y. Kuang, Z. Ge, *Adv. Mater.* **2018**, *30*, 1703005.
[3] J. Zhang, C. Yan, W. Wang, Y. Xiao, X. Lu, S. Barlow, T. C. Parker, X. Zhan, S. R. Marder, *Chem. Mater.* **2018**, DOI: 10.1021/acs.chemmater.7b04499.
[4] X. Ma, Y. Mi, F. Zhang, Q. An, M. Zhang, Z. Hu, X. Liu, J. Zhang, W. Tang, *Adv. Energy Mater.*, **2018**, 1702854.
[5] R. Yu, S. Zhang, H. Yao, B. Guo, S. Li, H. Zhang, M. Zhang, J. Hou, *Adv. Mater.* **2017**, *29*, 1700437.
[6] W. Su, Q. Fan, X. Guo, X. Meng, Z. Bi, W. Ma, M. Zhang, Y. Li, *Nano Energy* **2017**, *38*, 510.
[7] H. Yao, Y. Cui, R. Yu, B. Gao, H. Zhang, J. Hou, *Angew. Chem. Int. Ed.* **2017**, *56*, 3045.
[8] T. Liu, Y. Guo, Y. Yi, L. Huo, X. Xue, X. Sun, H. Fu, W. Xiong, D. Meng, Z. Wang, F. Liu, T. P. Russell, Y. Sun, *Adv. Mater.* **2016**, *28*, 10008.
[9] P. Cheng, M. Zhang, T.-K. Lau, Y. Wu, B. Jia, J. Wang, C. Yan, M. Qin, X. Lu, X. Zhan, *Adv. Mater.* **2017**, *29*, 1605216.
[10] L. Zhong, L. Gao, H. Bin, Q. Hu, Z.-G. Zhang, F. Liu, T. P. Russell, Z. Zhang, Y. Li, *Adv. Energy Mater.* **2017**, *7*, 1602215.
[11] Z. Li, X. Xu, W. Zhang, X. Meng, Z. Genene, W. Ma, W. Mammo, A. Yartsev, M. R. Andersson, R. A. J. Janssen, E. Wang, *Energy Environ. Sci.* **2017**, *10*, 2212.
[12] D. Baran, R. S. Ashraf, D. A. Hanifi, M. Abdelsamie, N. Gasparini, J. A. Rohr, S. Holliday, A. Wadsworth, S. Lockett, M. Neophytou, C. J. M. Emmott, J. Nelson, C. J. Brabec, A. Amassian, A. Salleo, T. Kirchartz, J. R. Durrant, I. McCulloch, *Nat. Mater.* **2017**, *16*, 363.
[13] P. Bi, F. Zheng, X. Yang, M. Niu, L. Feng, W. Qin, X. Hao, *J. Mater. Chem. A* **2017**, *5*, 12120.
[14] W. Su, Q. Fan, X. Guo, B. Guo, W. Li, Y. Zhang, M. Zhang, Y. Li, *J. Mater. Chem. A* **2016**, *4*, 14752.
[15] W. Zhao, S. Li, S. Zhang, X. Liu, J. Hou, *Adv. Mater.* **2017**, *29*, 1604059.
[16] T. Kumari, S. M. Lee, S.-H. Kang, S. Chen, C. Yang, *Energy Environ. Sci.* **2017**, *10*, 258.
[17] T. Zhang, X. Zhao, D. Yang, Y. Tian, X. Yang, *Adv. Energy Mater.*, 1701691.
[18] B. Xia, L. Yuan, J. Zhang, Z. Wang, J. Fang, Y. Zhao, D. Deng, W. Ma, K. Lu, Z. Wei, *J. Mater. Chem. A* **2017**, *5*, 9859.
[19] L. Nian, K. Gao, F. Liu, Y. Kan, X. Jiang, L. Liu, Z. Xie, X. Peng, T. P. Russell, Y. Ma, *Adv. Mater.* **2016**, *28*, 8184.
[20] L. Nian, K. Gao, Y. Jiang, Q. Rong, X. Hu, D. Yuan, F. Liu, X. Peng, T. P. Russell, G. Zhou, *Adv. Mater.* **2017**, *29*, 1700616.
[21] N. Gasparini, L. Lucera, M. Salvador, M. Prosa, G. D. Spyropoulos, P. Kubis, H.-J. Egelhaaf, C. J. Brabec, T. Ameri, *Energy Environ. Sci.* **2017**, *10*, 885.
[22] F. Zhao, Y. Li, Z. Wang, Y. Yang, Z. Wang, G. He, J. Zhang, L. Jiang, T. Wang, Z. Wei, W. Ma, B. Li, A. Xia, Y. Li, C. Wang, *Adv. Energy Mater.* **2017**, *7*, 1602552.
[23] H. Zhang, X. Wang, L. Yang, S. Zhang, Y. Zhang, C. He, W. Ma, J. Hou, *Adv. Mater.*, **2017**, *29*, 1703777.
[24] G. Zhang, K. Zhang, Q. Yin, X.-F. Jiang, Z. Wang, J. Xin, W. Ma, H. Yan, F. Huang, Y. Cao, *J. Am. Chem. Soc.* **2017**, *139*, 2387.
[25] J. Zhang, Y. Zhang, J. Fang, K. Lu, Z. Wang, W. Ma, Z. Wei, *J. Am. Chem. Soc.* **2015**, *137*, 8176.

[26] H. Lu, J. Zhang, J. Chen, Q. Liu, X. Gong, S. Feng, X. Xu, W. Ma, Z. Bo, *Adv. Mater.* **2016**, *28*, 9559.
[27] M. Zhang, F. Zhang, Q. An, Q. Sun, W. Wang, X. Ma, J. Zhang, W. Tang, *J. Mater. Chem. A* **2017**, *5*, 3589.
[28] Q. An, F. Zhang, X. Yin, Q. Sun, M. Zhang, J. Zhang, W. Tang, Z. Deng, *Nano Energy* **2016**, *30*, 276.
[29] T. Liu, X. Xue, L. Huo, X. Sun, Q. An, F. Zhang, T. P. Russell, F. Liu, Y. Sun, *Chem. Mater.* **2017**, *29*, 2914.
[30] Y. Chen, P. Ye, Z.-G. Zhu, X. Wang, L. Yang, X. Xu, X. Wu, T. Dong, H. Zhang, J. Hou, F. Liu, H. Huang, *Adv. Mater.* **2017**, *29*, 1603154.
[31] T. Liu, L. Huo, X. Sun, B. Fan, Y. Cai, T. Kim, J. Y. Kim, H. Choi, Y. Sun, *Adv. Energy Mater.* **2016**, *6*, 1502109.
[32] L. Lu, W. Chen, T. Xu, L. Yu, *Nat. Commun.* **2015**, *6*, 7327.
[33] T. H. Lee, M. A. Uddin, C. Zhong, S.-J. Ko, B. Walker, T. Kim, Y. J. Yoon, S. Y. Park, A. J. Heeger, H. Y. Woo, J. Y. Kim, *Adv. Energy Mater.* **2016**, *6*, 1600637.
[34] V. Gupta, V. Bharti, M. Kumar, S. Chand, A. J. Heeger, *Adv. Mater.* **2015**, *27*, 4398.
[35] N. Gasparini, X. Jiao, T. Heumueller, D. Baran, G. J. Matt, S. Fladischer, E. Spiecker, H. Ade, C. J. Brabec, T. Ameri, *Nat. Energy* **2016**, *1*, 16118.
[36] L. Lu, T. Xu, W. Chen, E. S. Landry, L. Yu, *Nat. Photon.* **2014**, *8*, 716.
[37] B. Fan, W. Zhong, X.-F. Jiang, Q. Yin, L. Ying, F. Huang, Y. Cao, *Adv. Energy Mater.* **2017**, *7*, 1602127.
